# Pivotal interplays between fecal metabolome and gut microbiome reveal functional signatures in cerebral ischemic stroke

**DOI:** 10.1186/s12967-022-03669-0

**Published:** 2022-10-08

**Authors:** Lanlan Zhao, Cheng Wang, Shanxin Peng, Xiaosong Zhu, Ziyi Zhang, Yanyan Zhao, Jinling Zhang, Guoping Zhao, Tao Zhang, Xueyuan Heng, Lei Zhang

**Affiliations:** 1 Department of Biostatistics, School of Public Health, Cheeloo College of Medicine, Shandong University, Jinan, 250003 China; 2 Microbiome-X, National Institute of Health Data Science of China, Cheeloo College of Medicine, Shandong University, Jinan, 25000 China; 3 Department of Neurology, Lin Yi People’s Hospital, Shandong University, Linyi, China; 4 State Key Laboratory of Microbial Technology, Shandong University, Qingdao, China; 5 CAS Key Laboratory of Computational Biology, Bio-Med Big Data Center, Shanghai Institute of Nutrition and Health, University of Chinese Academy of Sciences, Chinese Academy of Sciences, Shanghai, China

**Keywords:** Metabolomics, Microbiome, Integrative analysis, Gut microbiota, Ischemic stroke

## Abstract

**Background:**

Integrative analysis approaches of metagenomics and metabolomics have been widely developed to understand the association between disease and the gut microbiome. However, the different profiling patterns of different metabolic samples in the association analysis make it a matter of concern which type of sample is the most closely associated with gut microbes and disease. To address this lack of knowledge, we investigated the association between the gut microbiome and metabolomic profiles of stool, urine, and plasma samples from ischemic stroke patients and healthy subjects.

**Methods:**

We performed metagenomic sequencing (feces) and untargeted metabolomics analysis (feces, plasma, and urine) from ischemic stroke patients and healthy volunteers. Differential analyses were conducted to find key differential microbiota and metabolites for ischemic stroke. Meanwhile, Spearman’s rank correlation and linear regression analyses were used to study the association between microbiota and metabolites of different metabolic mixtures.

**Results:**

Untargeted metabolomics analysis shows that feces had the most abundant features and identified metabolites, followed by urine and plasma. Feces had the highest number of differential metabolites between ischemic stroke patients and the healthy group. Based on the association analysis between metagenomics and metabolomics of fecal, urine, and plasma, fecal metabolome showed the strongest association with the gut microbiome. There are 1073, 191, and 81 statistically significant pairs (*P* < 0.05) in the correlation analysis for fecal, urine, and plasma metabolome. Fecal metabolites explained the variance of alpha-diversity of the gut microbiome up to 31.1%, while urine and plasma metabolites only explained the variance of alpha-diversity up to 13.5% and 10.6%. Meanwhile, there were more significant differential metabolites in feces than urine and plasma associated with the stroke marker bacteria.

**Conclusions:**

The systematic association analysis between gut microbiome and metabolomics reveals that fecal metabolites show the strongest association with the gut microbiome, followed by urine and plasma. The findings would promote the association study between the gut microbiome and fecal metabolome to explore key factors that are associated with diseases. We also provide a user-friendly web server and a R package to facilitate researchers to conduct the association analysis of gut microbiome and metabolomics.

**Supplementary Information:**

The online version contains supplementary material available at 10.1186/s12967-022-03669-0.

## Background

The human gut microbiome plays a key role in understanding the etiology of diseases such as hypertension, diabetes, and cardiovascular diseases [1–4]. The dynamic changes of certain gut bacteria are associated with the changes of clinical blood markers and metabolites [5–7]. Metabolomics profiles the qualitative and quantitative state of small molecules, such as amino acids in complex metabolic mixtures, and is inherently associated with the microbiome [8, 9]. For example, the fecal metabolome was specifically regarded as a direct readout of metabolic functions of gut microbiota [10]. Cooperative alternations of the gut microbiome and host metabolome provide integrated information to elucidate the role of gut microbiota and metabolites in the disrupted pathways [11, 12].

Integrative analysis of microbiome and metabolomics has been widely adopted as an effective strategy to understand the association between health outcomes and microbiome [13, 14]. Metabolomes from different biological sample sources have distinct profiles and show different extent of associations with the gut microbiome [8, 15, 16]. Therefore, the choice of different metabolic samples in the association analysis is critical to investigate the interplay between gut microbiota and host metabolites. For example, the TwinsUK cohort study revealed that gut microbiome and fecal metabolites were strongly associated because 90% of the bacteria species were associated with 82% of the fecal metabolites, while only 34% of the bacteria species showed association with 24% of the blood metabolites [8]. A nutrition intervention study showed that the gut microbiome and the fecal metabolome remained significantly perturbed even after the urine and plasma metabolomes recovered their balance [16].

Stroke is one of the most prevalent cardiovascular diseases, which is the second leading cause of death and a major cause of disability worldwide [17, 18]. Cerebral ischemic stroke (CIS) caused by disruption of brain blood flow leads to permanent neurological deficits, dementia, and death [19]. Recent studies demonstrated that significant dysbiosis of the gut microbiota occurred in CIS patients. For example, CIS patients had more opportunistic pathogens, such as *Enterobacter*, *Megasphaera*, *Oscillibacter*, and *Desulfovibrio*, and fewer commensal or beneficial genera including *Bacteroides*, *Prevotella*, and *Faecalibacterium* [20, 21]. Meanwhile, several studies showed that stroke patients had low short chain fatty acids (SCFAs) levels in feces [21] and high trimethylamine oxide (TMAO) level in plasma [22–24], suggesting that the metabolic dysregulation is associated with the pathogenic mechanism of CIS. Moreover, the association analysis of gut microbiome and metabolome revealed ischemic stroke related microbes were associated with characteristic metabolites. For instance, integrated 16S rRNA gene sequencing and metabolomics analysis of plasma showed that *Proteobacteria* was positively correlated with L-phenylalanine, while it was negatively correlated with eicosapentaenoic acid (EPA), which might serve as potential diagnostic and therapeutic markers for ischemic stroke [25].

To evaluate which type of metabolome from various metabolic mixtures is more closely related to the fecal metagenome, we present a heuristic method to investigate the associations between fecal metagenome and various metabolomes of fecal, urine, and blood samples in CIS patients and healthy subjects. The association between metagenome and metabolome is evaluated based on the Spearman’s rank correlation and linear regression model. A user-friendly web server is developed to allow researchers to conduct the association analysis and visualization efficiently. Our study reveals that different metabolic mixtures (plasma, urine, and feces) show characteristic metabolic dysregulation associated with CIS. Fecal metabolome shows the strongest association with metagenome. More importantly, a substantial number of fecal metabolites are associated with bacteria species related to CIS.

## Methods

### Study subjects

A total of 60 subjects were recruited for this study. 30 patients with CIS were enrolled from Qilu Hospital of Shandong University between May 2017 and January 2018. They had been diagnosed by skull computed tomography examination. And 30 CIS patients did not suffer from any pre-existing metabolic or gut disease. In addition, 30 healthy volunteers who were examined to ensure that they had no metabolic, cardiovascular or cerebrovascular diseases or cancer were included as the standard control group. All these individuals did not receive any antibiotics or probiotics at least one month prior to the collection of biospecimens.

### Clinical measurements

Blood samples were gathered from patients with stroke at admission and healthy subjects at the physical examination center. Serum levels of low-density lipoprotein (LDL), the glucose of blood (GLU), high-density lipoprotein (HDL), uric acid (UA), triglycerides (TG), and homocysteine (HCY) were measured using standard techniques.

### Sample collection for metagenomics and metabolomics

The stool samples freshly collected from each participant were immersed in absolute ethyl alcohol, transported to the laboratory with ice pack as soon as possible, and finally stored at − 80 °C freezer. The morning urine was collected, and each 1 mL of urine was mixed with 50μL of 0.42% sodium azide preservative. The pH of urine was adjusted to 7.0 with 1 M Tris–HCl (pH 7.0) and stored at − 80 °C freezer. The morning fasting blood was collected and centrifuged to obtain the plasma, which was stored at − 80 °C freezer for metabolomics analysis.

### Statistical analysis of clinical data

Descriptive statistics, including mean (standard deviation: SD) or median (interquartile range: IQR) for quantitative variables and percentage for categorical variables, were calculated to examine the baseline characteristics. Differences in clinical indices among groups were determined using χ^2^ test and Student’s *t*-test or Wilcoxon Rank-Sum tests for categorical and quantitative variables, respectively. Crude odds ratios and their 95% confidence intervals were estimated using univariate logistic regression models.

### Metagenomics data analysis

Alpha-diversity and beta-diversity analyses were performed to estimate the diversity of microbial taxa in both CIS and the control group. The richness and abundance of alpha-diversity for each group were calculated using the MicrobiotaProcess package in R, which were subject to statistical comparison and visualization using Wilcoxon Rank-Sum tests and ggplot2. The gut microbiota composition for each group was calculated based on Bray–Curtis dissimilarities at the species level and visualized using Principal Coordinates Analysis (PCoA). The statistically significant differential species were evaluated by the linear discriminant analysis of effect size (LEfSe) analysis using the Huttenhower lab Galaxy server (http://huttenhowersphharvard.edu/galaxy/). Functional annotation of microbial taxa was performed using the KEGG Orthology (KO) database. KEGG pathways at Level 3 were enriched, followed by statistical analysis using Statistical Analysis of Metagenomic Profiles (STAMP).

### Metabolomics data analysis

Differences in feces, urine, and plasma metabolites between the CIS patients and the control group were evaluated by Wilcoxon Rank-Sum tests and fold change values. The differentially expressed metabolites were mapped onto metabolic pathways using the KEGG database. The enriched pathways were counted only if more than three metabolites were mapped, and the corresponding enrichment factors were calculated.

### Construction of gut bacterium ecological network

The gut bacterium ecological network was constructed by calculating the Spearman’s rank correlation coefficient between microbiome species using Hmisc in R and visualized using Cytoscape (3.9.0) [26]. Only species that showed statistical differences were included in the network. Additional file 1: Figure S1 depicts the flowchart with a series of procedures to include the microbiome species. In the network, each edge denotes a significant correlation between a pair of species (*P* < 0.05 and |r|> 0.5). The size of a node is proportional to the number of significant interactions between species, and the color of a node indicates the phylum taxonomy.

### Association analysis across clinical, metagenomics and metabolomics data

Extensive association analyses were performed to evaluate the correlation between blood clinical indices, metagenomics, and metabolomics data. For clinical indices, the following six indices were included, namely, triglycerides (TG), low-density lipoprotein (LDL), uric acid (UA), glucose (GLU), homocysteine (HCY), and high-density lipoprotein (HDL). For metagenomics data, 725 statistically different species between CIS and control group were included. For metabolites, all identified metabolites were included. Spearman’s rank correlation coefficient was calculated by Hmisc package and visualized by heatmap using pheatmap packeage in R. Linear regression model was applied to assess the individual relationship between each metabolite and Chao1 or Shannon diversity.

### R package and web server

CorHeat, an open-source R package, was developed to embed the association analysis pipeline in the study (https://github.com/zllxm/CorHeat). A user-friendly web server called CorHeat Lab was developed that is capable of conducting the association analysis online (https://corheat-v1.shinyapps.io/CorHeat-v1/).

### Fecal DNA extraction and metagenome sequencing

The fecal DNA was extracted from fecal samples by the beadbeating method using a GNOME DNA Isolation Kit (MP Biomedicals). Approximately 1 μg DNA per sample was sonicated to fragments with a size of 350 bp. All the DNA fragments were end-polished, A-tailed, and ligated with the full-length adaptor for Illumina sequencing with further PCR amplification. The PCR products were purified using AMPure XP system. Libraries were analyzed for size distribution by Agilent2100 Bioanalyzer and quantified using real-time PCR. The clustering of the index-coded samples was performed on a cBot Cluster Generation System. After cluster generation, the library preparations were sequenced on an Illumina HiSeq platform and paired-end (PE) reads were generated.

### Metagenomic data preprocessing

Raw sequence reads were trimmed using Trimmomatic [27] to remove adapters and low-quality reads. The contaminating human reads were removed using Bowtie2 [28]. High-quality reads were assembled into contigs using MEGAHIT [29]. Prediction of Open Reading Frame (ORF) was performed on the assembled contigs using MetaGeneMark [30]. The redundancy in the ORF predicted results were eliminated by CD-HIT [31] to construct the non-redundant gene catalog. The gene abundance in each sample was determined by aligning the reads against the gene catalog using Bowtie2 and counting the number of reads mapped to each gene. To estimate the taxonomic profiles and functional annotations of the gut microbial metagenome, the predicted genes were aligned against the NCBI-nr database [32] and protein sequences in the KEGG databases [33] using DIAMOND blastp [34].

### GC-TOF–MS Experiment

GC-TOF–MS analysis was performed using an Agilent 7890 gas chromatography (GC) system coupled with a Pegasus HT time-of-flight mass spectrometer (TOF–MS). The system utilized a DB-5MS capillary column coated with 5% diphenyl cross-linked with 95% dimethylpolysiloxane (30 m × 250 μm inner diameter, 0.25 μm film thickness; J&W Scientific, Folsom, CA, USA). A 1μL aliquot of the analyte was injected in splitless mode. Helium was used as the carrier gas, the front inlet purge flow was 3 mL min^−1^, and the gas flow rate through the column was 1 mL min^−1^. The initial temperature was kept at 50 °C for 1 min, then raised to 310 °C at a rate of 10 °C min^−1^, then kept for 10 min at 310 °C. The injection, transfer line, and ion source temperatures were 280, 280, and 250 °C, respectively. The energy was − 70 eV in electron impact mode. The mass spectrometry data were acquired in full-scan mode with the m/z range of 50–500 at a rate of 20 spectra per second after a solvent delay of 6.27 min.

### GC–MS data preprocessing

Chroma TOF 4.3X software of LECO Corporation and LECO-Fiehn Rtx5 database were used for peak picking, data baselines filtering and calibration of the baseline, peak alignment, deconvolution analysis, peak identification, and integration of the peak area. Peaks that were detected in less than 50% of the quality control samples were removed. Both mass spectrum match and retention index match were considered in metabolites identification.

## Results

### Fecal metabolome shows the most abundant metabolic features

**Fig. 1:**
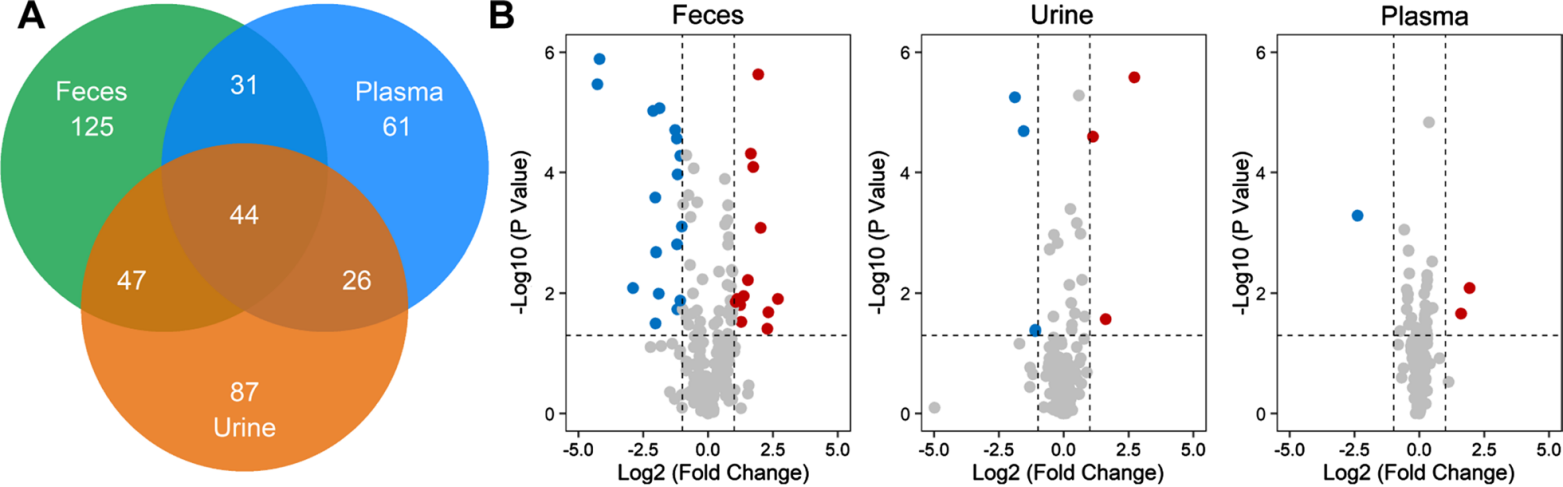
Metabolic profiling analysis of metabolic mixtures in feces, urine and plasma. **A** Venn diagram of number of identified metabolites in feces, urine, and plasma. **B** Volcano plots of metabolite changes of CIS versus control in feces, urine, and plasma. Each dot represents a metabolite identified in the sample. Blue dot represents a metabolite that is downregulated in the CIS. Red dot represents a metabolite that is upregulated in the CIS

The metabolic profiling of the three types of metabolic mixtures was performed on GC–MS platforms. After identification of metabolites, feces possess the most abundant features and identified metabolites, followed by urine and plasma. Also, the different mixtures show distinct and common features. Figure 1A shows the Venn diagram of the number of identified metabolites in three types of metabolic mixtures. There are 247, 204, and 162 metabolites identified in feces, urine, and plasma. We calculated the quantitative changes of metabolites with statistical tests between CIS and the control group in different types of mixtures (Fig. 1B). The numbers of differentially expressed metabolites (fold change > 2 or < 0.5, *P* < 0.05) are 30, 7, and 3 for feces, urine, and plasma, respectively. Therefore, fecal metabolites are more representative in the study of CIS-associated metabolome than urine and plasma. For example, among all disrupted metabolites, phenylacetic acid is identified in feces and up regulated in CIS group, which is the precursor metabolite for the generation of phenylacetylglutamine that is associated with cardiovascular disease (CVD) and incident major adverse cardiovascular events (myocardial infarction, stroke, or death) [35].

### Fecal metabolome reveals the strongest association with gut microbiota

**Fig. 2:**
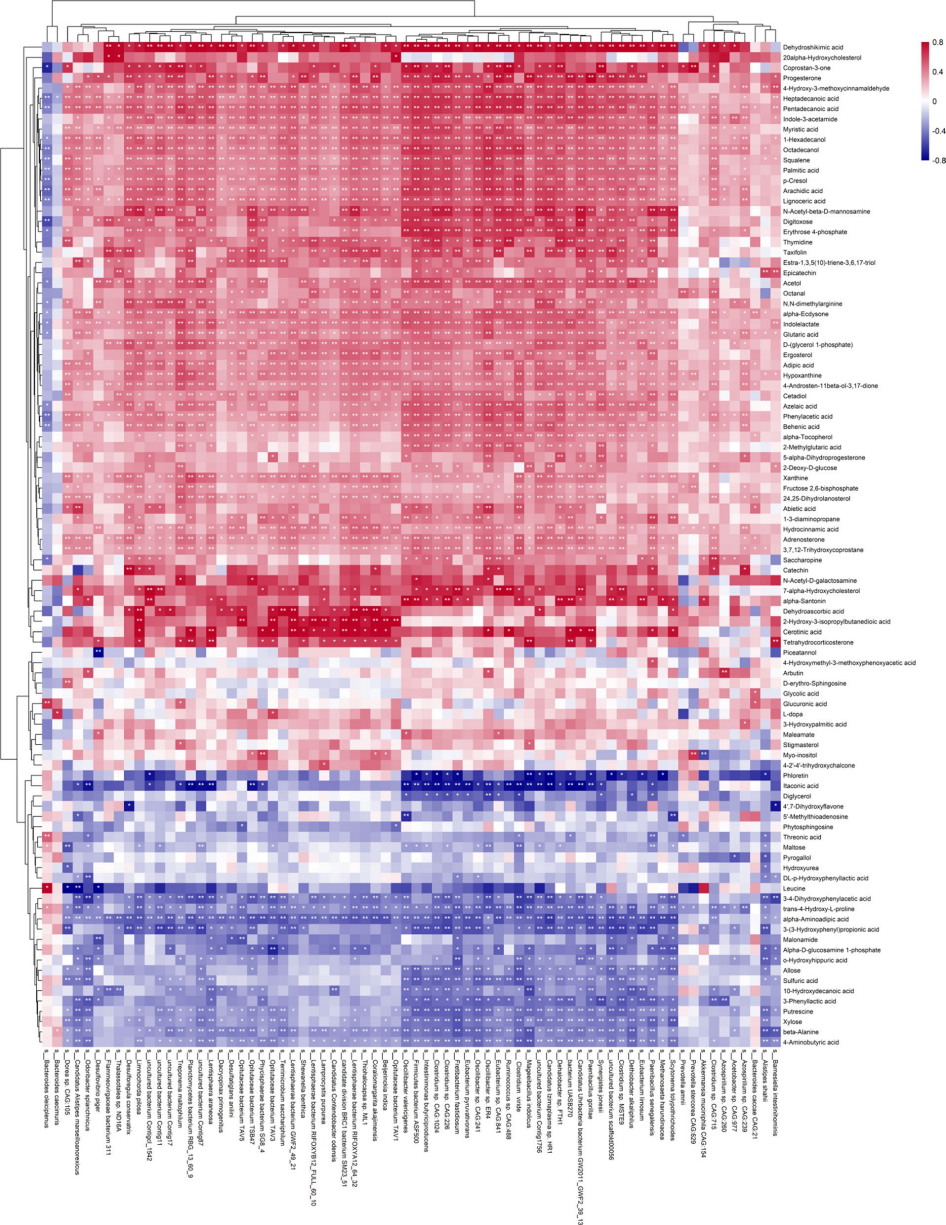
Heatmap of the Spearman’s rank correlation of species and fecal metabolites. 7056 pairs of correlations with 72 bacteria species and 98 fecal metabolites were plotted. Red squares indicate positive associations between these microbial species and clinical indexes. Blue squares indicate negative associations. The statistical significance was denoted inside the squares (*P < 0.05, **P < 0.01)

**Fig. 3:**
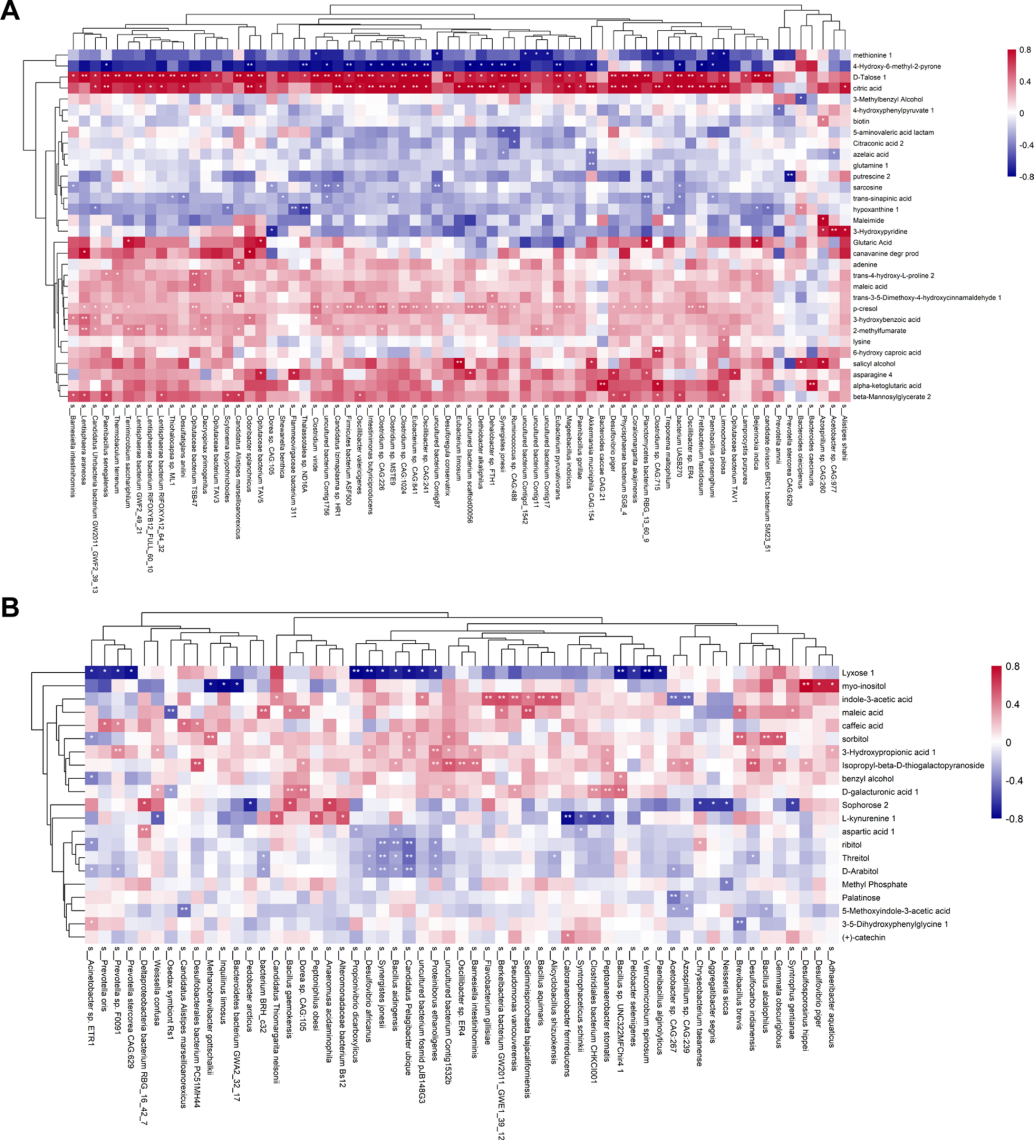
Heatmap of the Spearman’s rank correlation of species and urinary or plasma metabolites. Red squares indicate positive associations between these microbial species and clinical indexes. Blue squares indicate negative associations. The statistical significance was denoted inside the squares (*P < 0.05; **P < 0.01). **A** 2272 pairs of correlations with 71 bacteria species and 32 urinary metabolites were plotted. **B** 1197 pairs of correlations with 57 bacteria species and 21 plasma metabolites were plotted

The associations between the gut microbiome and the metabolome were evaluated using Spearman’s rank correlation analysis and linear regression model. The Spearman’s rank correlation coefficient was calculated for each pair of relative abundance of bacteria species and metabolites. A total of 725 statistically different bacteria species were included in the association analysis. Respectively, 247, 204, and 162 metabolites were considered in the correlation analysis for fecal, urine, and plasma metabolome. 1073 statistically significant pairs (*P* < 0.05) and 202 highly correlated pairs (|r|> 0.7, *P* < 0.05) were found between the gut microbiome and the fecal metabolome. 191 statistically significant pairs (*P* < 0.05) and 130 highly correlated pairs (|r|> 0.7, *P* < 0.05) were found between the gut microbiome and the urine metabolome. 81 statistically significant pairs (*P* < 0.05) and 16 highly correlated pairs (|r|> 0.7, *P* < 0.05) were found between the gut microbiome and the plasma metabolome. The correlation analysis results are clustered and visualized using heatmap in Fig. 2 and 3. By comparing the number of significant pairs and intensity of correlation, the fecal metabolome has the strongest correlation with the gut microbiome.

**Fig. 4:**
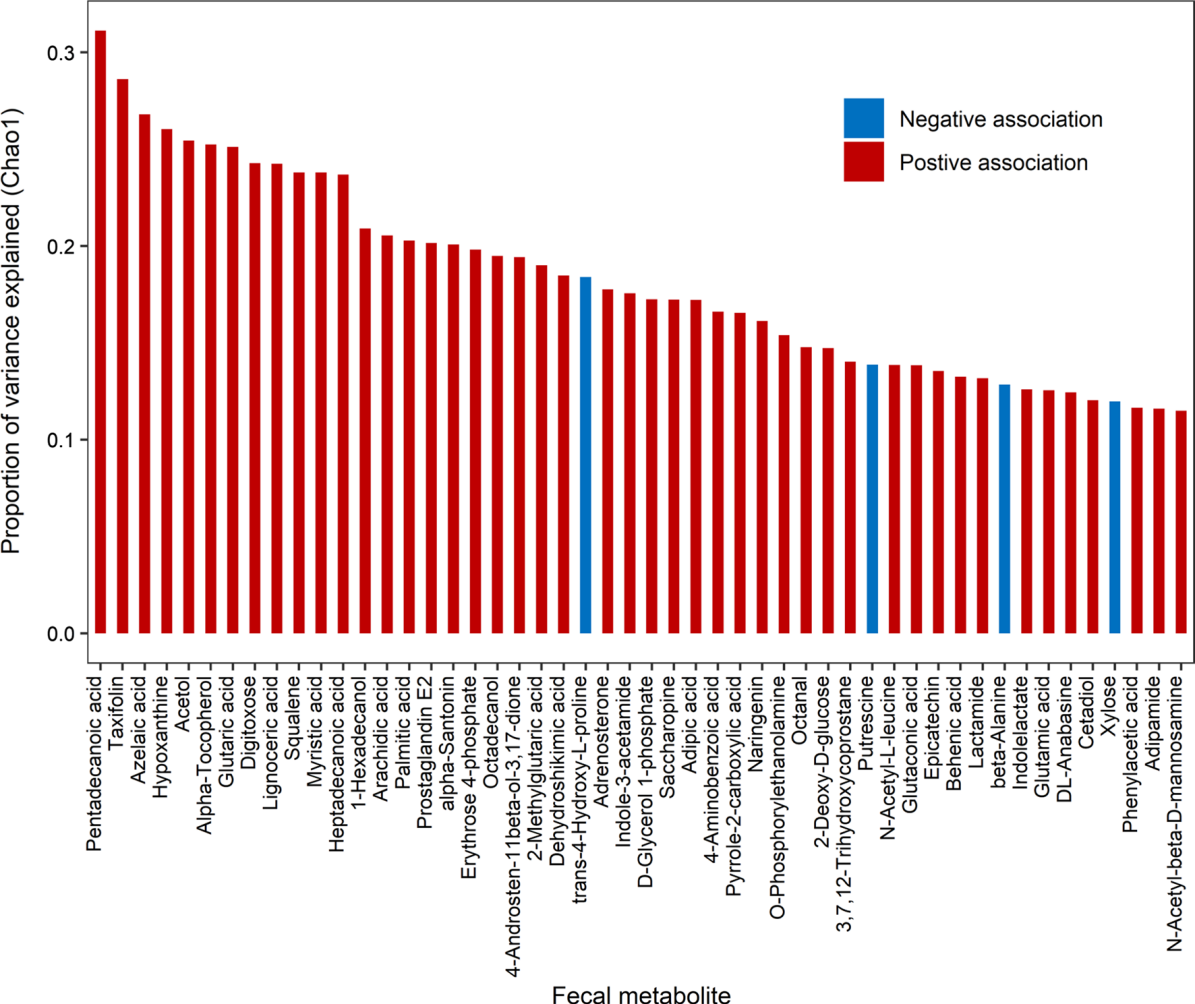
The proportion of variance in Chao1 diversity explained by each fecal metabolite. Red bar denotes positive associations between metabolite and Chao1 diversity, while blue bar denotes negative associations

To explore the association between metabolites and the alpha-diversity of the gut microbiota, linear regression analysis was performed for metabolites and alpha-diversity quantified by Chao1 and Shannon index. We estimated the proportion of variance in Chao1 and Shannon index explained by each metabolite. The results were shown in Fig. 4, Additional file 1: Figs. S11, and S13. It is found that 85 (feces), 7 (urine), and 5 (plasma) metabolites showed significant associations with the microbial diversity, besides, 58, 0, 0 of which remained significant after FDR correction. Moreover, 58 out of 85 fecal metabolites (FDR < 5%) explained a substantial proportion of the observed variance (> 10%) in the microbial diversity, and 16.7% (SD: 5.5%) of the observed variance on average, ranging from 10.0% for taurine to 31.1% for pentadecanoic acid. However, only 3 (urine) and 1 (plasma) metabolites explained up to 13.5% (p-cresol) and 10.6% (indole-3-acetic acid) of the observed variance on average in the microbial diversity. Consistent results were obtained for Shannon index, which is provided in supporting information. Therefore, fecal metabolome showed the highest association with the microbiome, followed by urine and plasma metabolome.

### Pivotal interplays of disrupted gut microbiota and fecal metabolites in CIS

**Fig. 5:**
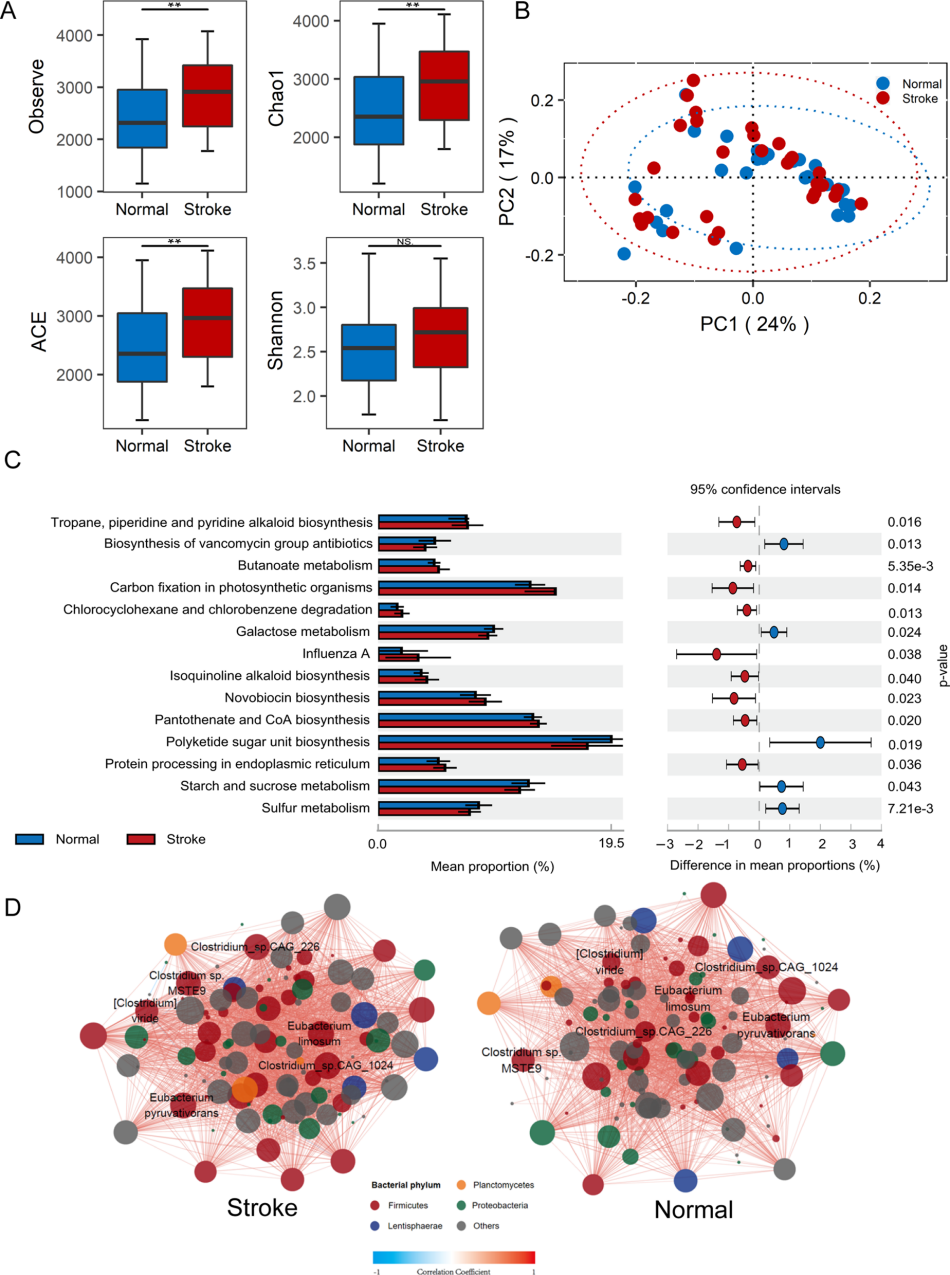
Gut microbiota taxonomic and functional comparison between CIS and the controls. **A** depicts the indices of alpha-diversity. **B** depicts the Principal Coordinates Analysis (PCoA) of beta-diversity. Each point represents a single sample in CIS and the controls. The two principal components (PC1 and PC2) explained 24% and 17%. **C** shows the relative abundance of KEGG pathways of functional annotations in the gut microbiota. The barplot with 95% confidence intervals denote the significantly different KEGG pathways between CIS and controls. **D** Gut bacterium-bacterium ecological network in CIS versus the controls. Correlations between taxa were calculated through Spearman’s rank correlation analysis. Statistical significance was determined for all pairwise comparisons. Only statistically significant correlations (*P* < 0.05) with |r|> 0.5 were plotted. The size of node, corresponding to individual microbial species, is proportional to the number of significant inter-species correlations. The color of node indicates the phylum to which the corresponding microbial species belong to. The color intensity of connective lines is proportional to the correlation coefficient, where blue lines indicate inverse correlations and red lines indicate positive correlations

**Fig. 6:**
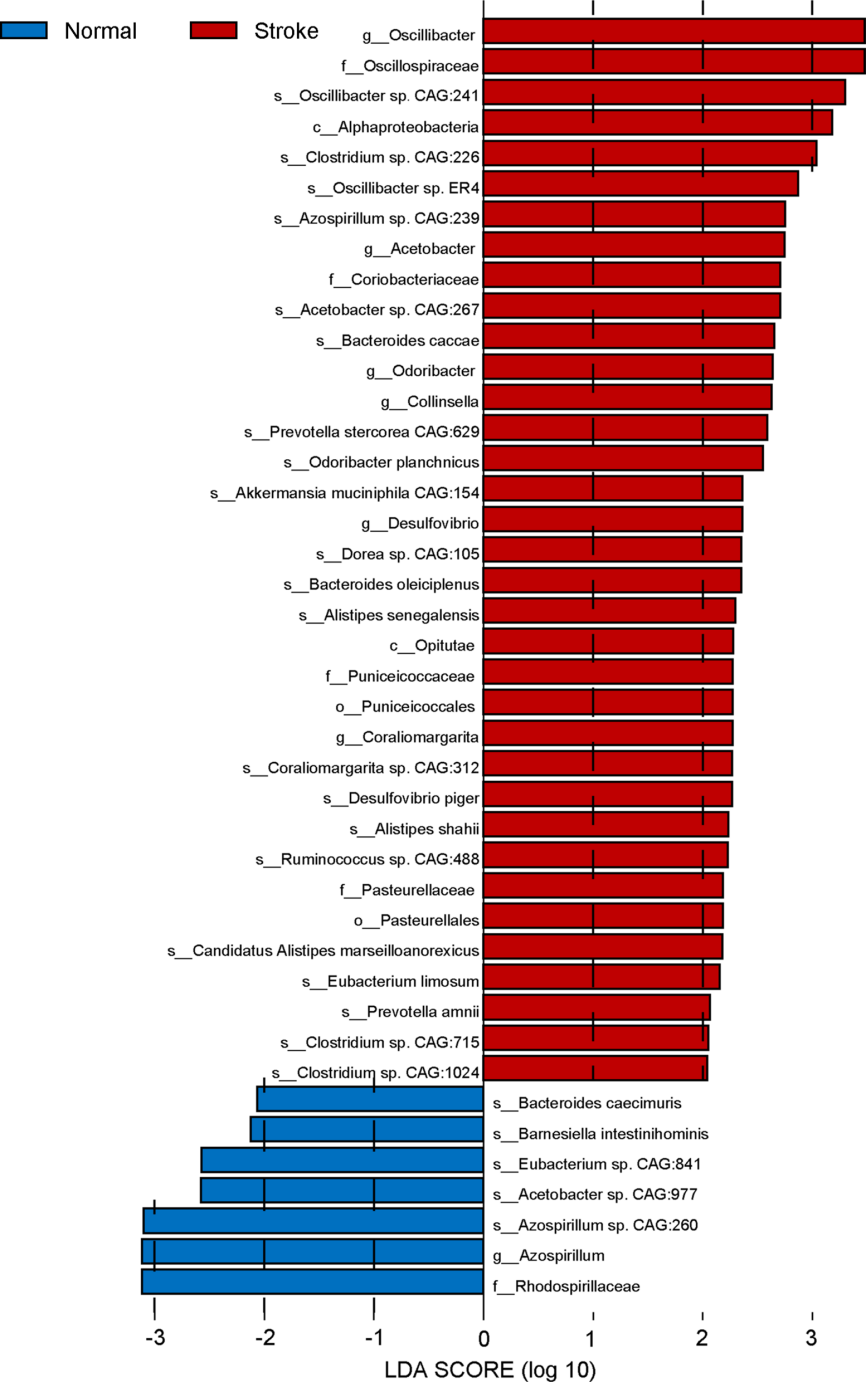
Histograms of significantly diferent abundant taxa with LDA score (log10) > 2.0 and *P* < 0.05

It is found that all microbial richness (Observe, Chao1, and Ace) and diversity (Shannon) were higher in the CIS group than the control group, though only microbial richness indices were statistically different (Fig. 5A). Based on the Principal Coordinates Analysis (PCoA) for beta-diversity analysis, there is no statistical difference in terms of microbial composition between CIS and the control group (Fig. 5B). Linear discriminant analysis effect size (LEfSe) analysis revealed that 33 different species and genera (26 species, 7 genera) were statistically different between the CIS and the control group (Fig. 6). We found that *Oscillibacter*, *Clostridium*, and SCFA producers such as *Odoribacter*, *Akkermansia*, and *Ruminococcus* were enriched in CIS group compared to the control group (Fig. 6). The KEGG pathway enrichment analysis at level 3 revealed that 14 metabolic pathways showed statistical differences (*P* < 0.05) between CIS and the control group (Fig. 5C). The ecological interaction analysis was performed to understand potential relationships among bacteria within the gut microbiota of CIS and the control group. As the ecological network shown in Fig. 5D, there was a stronger correlation in CIS compared with the control group. In the CIS group, bacteria *Oscillibacter sp. CAG:241* from the phylum *Firmicutes* showed the strongest interactions with species, and genus *Clostridium* showed the key and pillar role in bacteria interactions. Among the top 30 bacteria species based on the degrees of nodes, several species show stronger correlations in CIS than the control group, including *Oscillibacter sp. CAG:241*, *Clostridium sp. MSTE9*, *Clostridium viride*, *Clostridium sp. CAG:226*, and *Clostridium sp. CAG:1024*, which are associated with cardiovascular disease (CVD) and incident major adverse cardiovascular events (myocardial infarction, stroke, or death). Additionally, these *Clostridium* species enriched in CIS closely interacted with each other and formed a connected group in CIS. Such changes in the gut microbiome ecological network suggested that interspecies communication or interplay was significantly altered in CIS subjects.

**Fig. 7:**
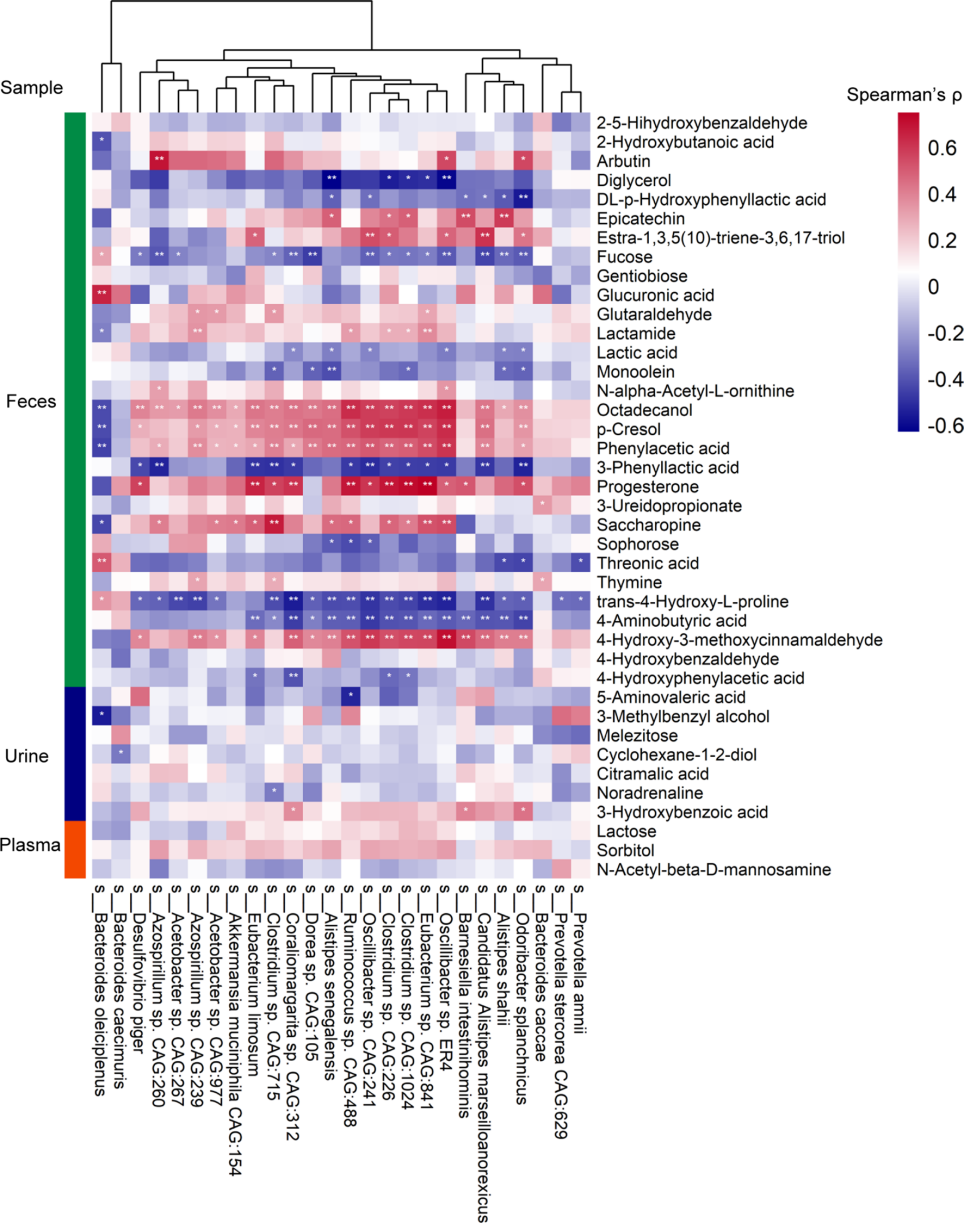
Heatmap of the Spearman’s rank correlation of significantly differential species and metabolites. The statistical significance was denoted inside the squares (*P < 0.05, **P < 0.01)

**Fig. 8:**
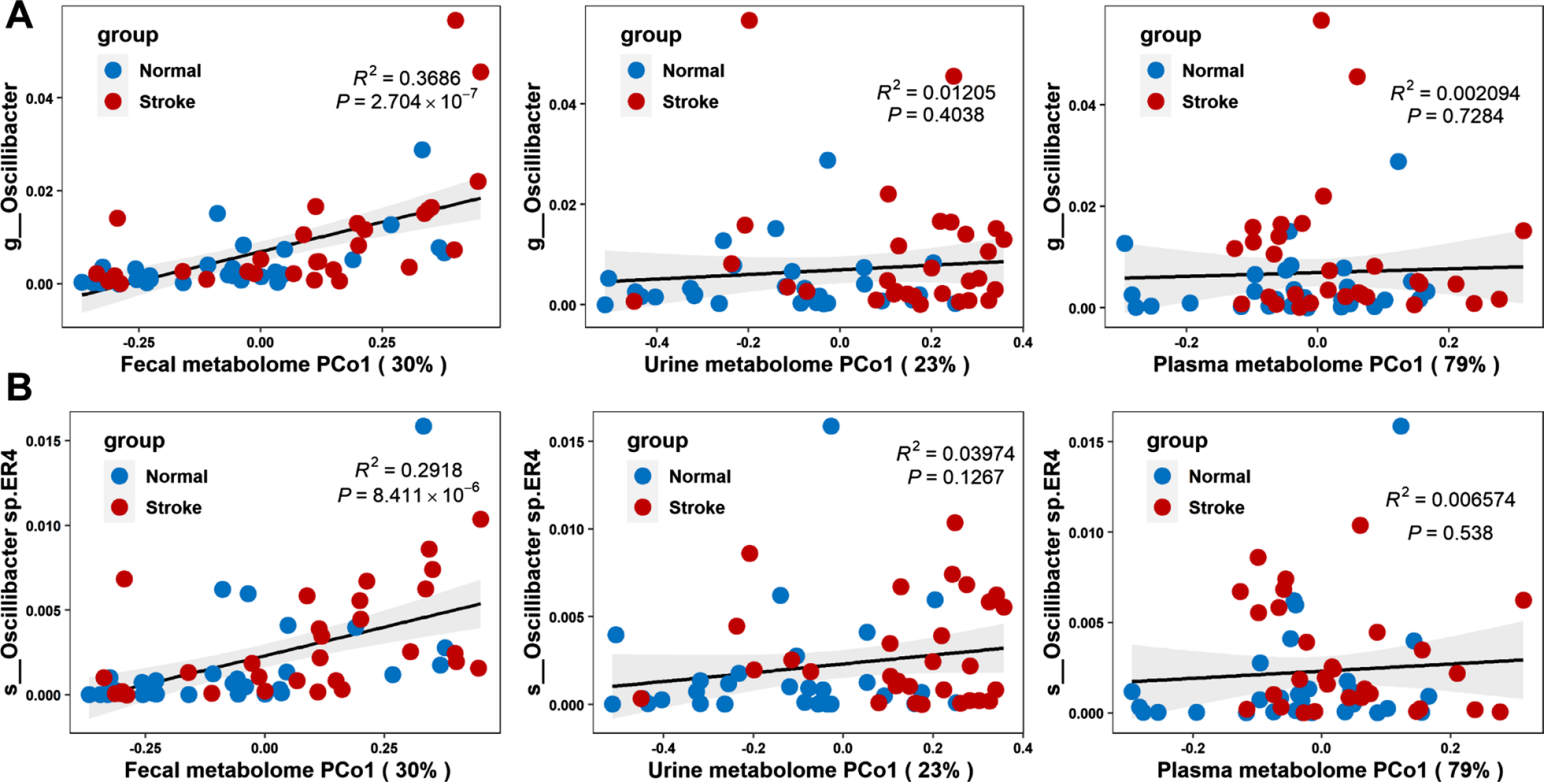
Association between bactriea data and the first principal coordinate (PCo1) of metabolomics data. R^2^ and its significance were calculated using the ischemic stroke and control samples together. The black line and gray area show a linear model and its 95% confidence interval describing the overall trend. **A** Correlation between *Oscillibacter* and the first principal coordinate (PCo1) of fecal, urine, and plasma metabolomics data. **B** Correlation between *Oscillibacter sp.ER4* and the first principal coordinate (PCo1) of fecal, urine, and plasma metabolomics data

We examined the association between the differential expressed metabolites and bacteria species in both CIS and control group. The Spearman’s rank correlation coefficient was calculated for each pair of relative abundance of differential bacteria species and metabolites. Here, only the most significant different metabolites were considered, which included 30, 7, and 3 metabolites for feces, urine, and plasma. Figure 7 shows the comparison of Spearman’s correlation between differential expressed metabolites and bacteria species. By comparing the number of significant pairs and intensity of correlation, the fecal metabolome had the strongest correlation with the gut microbiome, followed by the urine and plasma metabolome. Particularly, it is found that a fecal metabolite phenylacetic acid, the precursor substance of phenylacetyl glutamine (PAGln) that is related to CVD, was strongly associated with some bacteria species related to CIS, including *Oscillibacter sp. CAG:241* (r = 0.51, *P* < 0.001), *Clostridium viride* (r = 0.58, *P* < 0.001), *Clostridium sp.CAG: 1024* (r = 0.50, *P* < 0.001), *Clostridium sp.CAG: 226* (r = 0.48, *P* < 0.001), and *Clostridium sp.CAG: 715* (r = 0.40, *P* < 0.01). The linear regression analysis was also performed between individual bacteria species and all disrupted metabolites (Fig. 8 and Additional file 1: Figure S15). Fecal metabolites had the strongest association with the bacteria species. For instance, the fecal metabolome explained the 36.7% variance of *Oscillibacter*, while urine and plasma metabolome had no significance in the association analysis. Therefore, the study of CIS and the control group present that the fecal metabolome not only has the strongest association with the gut microbiome but also contributes the most to understanding the disrupted gut microbiota and metabolisms.

## Discussion

To investigate and evaluate the relationship between the gut microbiome and three sample types of metabolic mixtures, including feces, urine, and plasma, we performed a systematic analysis about the association between metagenomics and metabolomics data of the ischemic stroke and the control subjects. The integrative analysis of metagenomics and metabolomics reveals that fecal metabolome has the most abundant features with the strongest association with the gut microbiome in terms of bacteria diversity and abundance. The linear regression analyses showed that 58 fecal metabolites explained a substantial proportion of the observed variance (> 10%, up to 31.1%) of the microbial diversity. Compared with plasma and urine metabolome, fecal metabolome has the most significant number of differentially expressed metabolites between CIS and the control group. Furthermore, several disrupted fecal metabolites have strong correlations with the significantly differential bacteria species. These findings highlight the importance of fecal metabolites in metagenomic association analysis, which may serve as a reference for the selection of metabolic mixture in biomedical research.

The integrative analysis of gut microbiome and metabolome was applied to the real-world scenario for biomarker discovery in ischemic stroke. Fecal metabolome showed more significantly dysregulated metabolites between the CIS and the control group than urine and plasma. Phenylacetic acid, a significantly differential metabolite in the fecal metabolome, deserves special attention, because it is the precursor metabolite for generation of phenylacetylglutamine. The microbial gene *porA* facilitates dietary phenylalanine conversion into phenylacetic acid, with subsequent host generation of PAGln and phenylacetylglycine (PAGly) [35]. PAGln has been reported to be associated with cardiovascular disease (CVD) and incident major adverse cardiovascular events (myocardial infarction, stroke, or death) [35–37]. PAGln significantly impacted platelet function, accelerates platelet clot formation, and enhanced thrombosis potential in vivo [35]. Moreover, the disrupted fecal metabolites are associated with some disrupted bacteria species, and these disrupted bacteria species have been reported to be associated with cardiovascular disease, including *Oscillibacter* at the genus level and *Oscillibacter sp. CAG:241*, *Clostridium sp.CAG:715*, *Clostridium sp. CAG:226*, and *Clostridium sp. CAG:1024* [20, 38, 39].

It should be noted that due to the limited sample size of the study, some unexpected biases may exist, such as the confidence level of metabolite identification and biomarkers for ischemic stroke. Therefore, further improvements, such as increasing the sample size and designing studies of different diseases, would facilitate a more informative association map between gut microbiome and metabolomics. Overall, our study advances the knowledge that the fecal metabolites and gut microbiota are interconnected strongly and integrative analysis plays an important role in understanding the mechanisms of disease and biomarker discovery.

## Conclusions

Alternations of gut microbiome and metabolome provide integrated information to elucidate the role of gut microbiota and metabolites in understanding the mechanisms of disease. Different metabolic samples have distinct metabolic profiles and show different degree of associations with gut microbiome. Our integrative analysis of gut microbiome and metabolomics of feces, urine, and plasma reveal that fecal metabolites provide the most abundant metabolic information and show the strongest association with gut microbiome, which promotes the study of interplay of gut microbiota and metabolites to understand the disease. This study highlights the importance of fecal metabolites in metagenomic association analysis and provides a reference for appropriate metabolic sample selection in biomedical research. Furthermore, the integrative microbiome-metabolome association study with application to cerebral ischemic stroke shows that fecal metabolome provides comprehensive and informative metabolic status to advance the biomarker discovery in certain diseases. Understanding the interplay between fecal metabolites and gut microbiome will facilitate the multi-omics approach in biomedical and translational research, such as the development of personalized multimodal interventions for promoting health.

## Supplementary Information

**Additional file 1: Figure S1.** The selection criteria of bacteria for the ecological network analysis. **Figure S2.** Volcano plots of feature changes of CIS versus control in feces, urine, and plasma. **Figure S3.** Metabolic pathway enrichment of differential metabolites in feces. **Figure S4.** Metabolic pathway enrichment of differential metabolites in urine. **Figure S5.** Metabolic pathway enrichment of differential metabolites in plasma. **Figure S6.** Hierarchical clustered heatmap of the Spearman’s rank correlation coefficient of gut microbial species and blood clinical indexes. **Figure S7.** Hierarchical clustered heatmap of the Spearman’s rank correlation coefficient of fecal metabolites and blood clinical indexes. **Figure S8.** Hierarchical clustered heatmap of the Spearman’s rank correlation coefficient of urine metabolites and blood clinical indexes. **Figure S9.** Hierarchical clustered heatmap of the Spearman’s rank correlation coefficient of plasma metabolites and blood clinical indexes. **Figure S10.** The proportion of variance in Shannon diversity explained by fecal metabolites. **Figure S11.** The proportion of variance in Chao1 diversity explained by plasma metabolites. **Figure S12.** The proportion of variance in Shannon diversity explained by plasma metabolites. **Figure S13.** The proportion of variance in Chao1 diversity explained by urine metabolites. **Figure S14.** The proportion of variance in Shannon diversity explained by urine metabolites. **Figure S15.** Correlation between bacteria data and the first principal coordinate (PCo1) of fecal, urine, and plasma metabolomics data. **Figure S16.** Showcase of association analysis on the CorHeat Lab web server. **Table S1.** Characteristics of the study participants and result of univariate logistic regression. **Table S2.** Metabolites that differ significantly in each metabolic sample.

## Data Availability

The data supporting the findings of this study are available in the supplemental material. The R package CorHeat is publicly available at https://github.com/zllxm/CorHeat. The web server CorHeat Lab with example data is available at https://corheat-v1.shinyapps.io/CorHeat-v1/. The raw metagenomics data are available on The National Omics Data Encyclopedia (NODE) database (https://www.biosino.org/node, experiment ID: OEX016816). The raw metabolomics data are available on the metabolomics workbench (https://www.metabolomicsworkbench.org/).

## Abbreviations

CIS: Cerebral ischemic stroke
SCFAs: Short chain fatty acids
TMAO: Trimethylamine oxide
EPA: Eicosapentaenoic acid
LDL: Low-density lipoprotein
HDL: High-density lipoprotein
GLU: Glucose
UA: Uric acid
TG: Triglycerides
HCY: Homocysteine
SD: Standard deviation
IQR: Interquartile range
PCoA: Principal coordinates analysis
LEfSe: Linear discriminant analysis of effect size
KO: KEGG orthology
STAMP: Statistical analysis of metagenomic profiles
OLS: Ordinary Least Square
PE: Paired-end
ORF: Prediction of open reading frame
GC: Gas chromatography
TOF–MS: Time-of-flight mass spectrometer
CVD: Cardiovascular disease
PAGln: Phenylacetyl glutamine
PAA: Phenylacetic acid
Gln: Glutamine
