# Supplementary material for: Pivotal interplays between fecal metabolome and gut microbiome reveal functional signatures in cerebral ischemic stroke
Source: J Transl Med. 2022 Oct 8;20:459. doi: 10.1186/s12967-022-03669-0 (PMC9548195; doi:10.1186/s12967-022-03669-0)
Supplement: Supplementary file 1 — Additional file 1: Figure S1. The selection criteria of bacteria for the ecological network analysis. Figure S2. Volcano plots of feature changes of CIS versus control in feces, urine, and plasma. Figure S3. Metabolic pathway enrichment of differential metabolites in feces. Figure S4. Metabolic pathway enrichment of differential metabolites in urine. Figure S5. Metabolic pathway enrichment of differential metabolites in plasma. Figure S6. Hierarchical clustered heatmap of the Spearman’s rank correlation coefficient of gut microbial species and blood clinical indexes. Figure S7. Hierarchical clustered heatmap of the Spearman’s rank correlation coefficient of fecal metabolites and blood clinical indexes. Figure S8. Hierarchical clustered heatmap of the Spearman’s rank correlation coefficient of urine metabolites and blood clinical indexes. Figure S9. Hierarchical clustered heatmap of the Spearman’s rank correlation coefficient of plasma metabolites and blood clinical indexes. Figure S10. The proportion of variance in Shannon diversity explained by fecal metabolites. Figure S11. The proportion of variance in Chao1 diversity explained by plasma metabolites. Figure S12. The proportion of variance in Shannon diversity explained by plasma metabolites. Figure S13. The proportion of variance in Chao1 diversity explained by urine metabolites. Figure S14. The proportion of variance in Shannon diversity explained by urine metabolites. Figure S15. Correlation between bacteria data and the first principal coordinate (PCo1) of fecal, urine, and plasma metabolomics data. Figure S16. Showcase of association analysis on the CorHeat Lab web server. Table S1. Characteristics of the study participants and result of univariate logistic regression. Table S2. Metabolites that differ significantly in each metabolic sample. [file 12967_2022_3669_MOESM1_ESM.pdf]

Supplementary material

Pivotal interplays between fecal metabolome and gut microbiome reveal functional signatures in cerebral ischemic stroke

Lanlan Zhao [1, 2, #], Cheng Wang [1, 2, #], Shanxin Peng [3], Xiaosong Zhu [3], Ziyi Zhang [1, 2], Yanyan Zhao [1, 2], Jinling Zhang [3], Guoping Zhao [1, 2, 4, 5], Tao Zhang [1, 2, *], Xueyuan Heng [3, *], Lei Zhang [1, 2, 4, *]

1. Department of Biostatistics, School of Public Health, Cheeloo College of Medicine, Shandong University, Jinan, China
2. Microbiome-X, National Institute of Health Data Science of China, Cheeloo College of Medicine, Shandong University, Jinan, China
3. Department of Neurology, Lin Yi People's Hospital, Shandong University, Linyi, China
4. State Key Laboratory of Microbial Technology, Shandong University, Qingdao, China
5. CAS Key Laboratory of Computational Biology, Bio-Med Big Data Center, Shanghai Institute of Nutrition and Health, University of Chinese Academy of Sciences, Chinese Academy of Sciences, Shanghai, China

# These authors contributed equally to this work

*** Lead corresponding author**

Lei Zhang, PhD,

Professor & PI,

Director, Microbiome-X, National Institute of Health Data Science of China; & Department of Biostatistics, School of Public Health, Cheeloo College of Medicine,

Shandong University, Jinan, 250003, China

Email: zhanglei7@sdu.edu.cn

OUTLINE

Figure S1. The selection criteria of bacteria for the ecological network analysis.

Figure S2. Volcano plots of feature changes of CIS versus control in feces, urine, and plasma.

Figure S3. Metabolic pathway enrichment of differential metabolites in feces.

Figure S4. Metabolic pathway enrichment of differential metabolites in urine.

Figure S5. Metabolic pathway enrichment of differential metabolites in plasma.

Figure S6. Hierarchical clustered heatmap of the Spearman's rank correlation coefficient of gut microbial species and blood clinical indexes.

Figure S7. Hierarchical clustered heatmap of the Spearman's rank correlation coefficient of fecal metabolites and blood clinical indexes.

Figure S8. Hierarchical clustered heatmap of the Spearman's rank correlation coefficient of urine metabolites and blood clinical indexes.

Figure S9. Hierarchical clustered heatmap of the Spearman's rank correlation coefficient of plasma metabolites and blood clinical indexes.

Figure S10. The proportion of variance in Shannon diversity explained by fecal metabolites.

Figure S11. The proportion of variance in Chao1 diversity explained by plasma metabolites.

Figure S12. The proportion of variance in Shannon diversity explained by plasma metabolites.

Figure S13. The proportion of variance in Chao1 diversity explained by urine metabolites.

Figure S14. The proportion of variance in Shannon diversity explained by urine metabolites.

Figure S15. Correlation between bacteria data and the first principal coordinate (PCo1) of fecal, urine, and plasma metabolomics data.

Figure S16. Showcase of association analysis on the CorHeat Lab web server

Table S1. Characteristics of the study participants and result of univariate logistic regression.

Table S2. Metabolites that differ significantly in each metabolic sample.

Baseline characteristics of study subjects

Table S1 shows the demographic characteristics and blood clinical test results of total 60 participants. There were no statistically significant differences in gender, age, triglycerides (TG), low-density lipoprotein (LDL), uric acid (UA), glucose (GLU), and homocysteine (HCY) between the CIS and control group. Since high-density lipoprotein (HDL) is a known key factor that is associated with CIS, the HDL in the CIS group was lower than the control group (1.10 ±0.19 vs. 1. 29 ±0.24, $P < 0.01$). By performing univariate logistic regression between clinical characteristics and outcomes, HDL was a protective factor that is significantly associated with the CIS group.

Associations between gut microbiome and blood clinical indices

To evaluate the correlations between gut microbiome and the blood clinical indices including TG, LDL, HDL, UA, GLU, and HCY, Spearman's rank correlation coefficient was calculated for each pair of relative abundance of bacterial species and blood clinical index. The result is clustered and visualized using heatmap in Figure S6. Some statistically significant correlations are observed for HDL, LDL, and GLU. For HDL, a few bacterial species have significant negative correlations, including *Prevotella stercorea CAG:629* (r = -0.38, $P < 0.01$), *Prevotella amnii* (r = -0.41, $P < 0.01$), *Acinetobacter sp. ETR1*, *Weissella confusa*, and *Bacteroides caccae CAG:21*. The LDL was negative correlated with *Dorea sp.CAG:105* (r = -0.27, $P < 0.05$) and *Caloranaerobacter ferrireducens*. GLU was negative correlated with *Akkermansia muciniphila* CAG:154 (r = -0.33, $P < 0.05$).

Associations between metabolome and blood clinical indices

To further explore the correlation between metabolome and the blood clinical indices, Spearman's rank correlation coefficient was calculated for each pair of metabolites and blood clinical index. The result is shown in Figure S7, 8, and 9. Plasma and urine

metabolome show a stronger correlation with blood clinical indices with 73 and 77 significant pairs (|r| >= 0.5, $P < 0.05$), while the fecal metabolome shows 52 significant pairs.

Proportion of variance in Shannon diversity explained by metabolites

To explore the association between metabolites and the abundance of the gut microbiota, ordinary least square (OLS) regression analysis was performed for metabolites and alpha-diversity quantified by Shannon index. We estimated the proportion of variance in Shannon index explained by each metabolite. The result is shown in FigureS10, S12, and S14. It is found that 77 (feces), 5 (urine), and 5 (plasma) metabolites showed significant associations with the microbial diversity, 48, 0, 0 of which remained significant after FDR correction. Moreover, 48 out of 77 fecal metabolites (FDR < 5%) explained a substantial proportion of the observed variance (>10%) in the microbial diversity. 17.8% (SD: 6.2%) of the observed variance on average, ranging from 10.2% for taurine to 32.6% for Azelaic acid. However, urine and plasma metabolites explained up to 11.9% (3-Hydroxyphenylacetic acid) and 9.1% (Indole-3-acetic acid) of the observed variance.

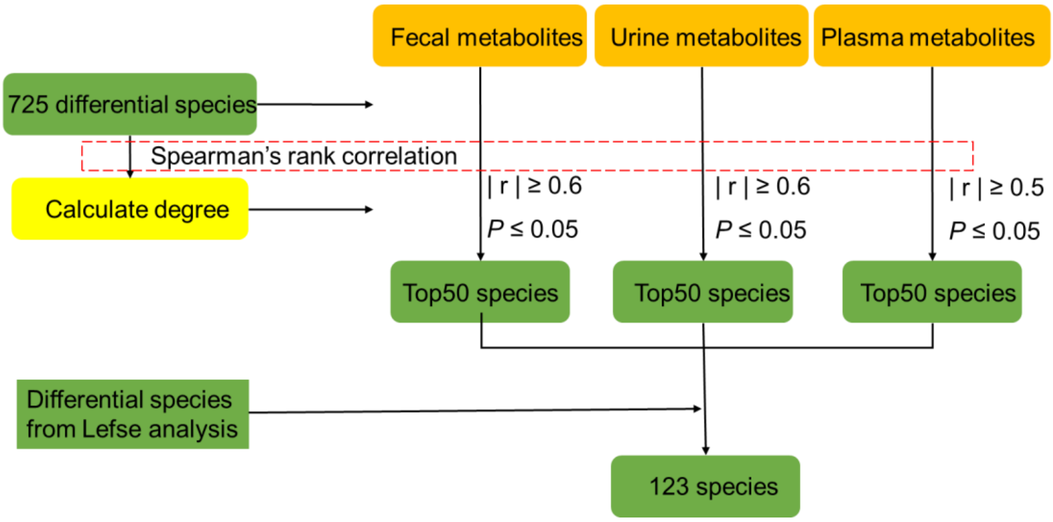

Figure S1. The selection criteria of bacteria for the ecological network analysis.

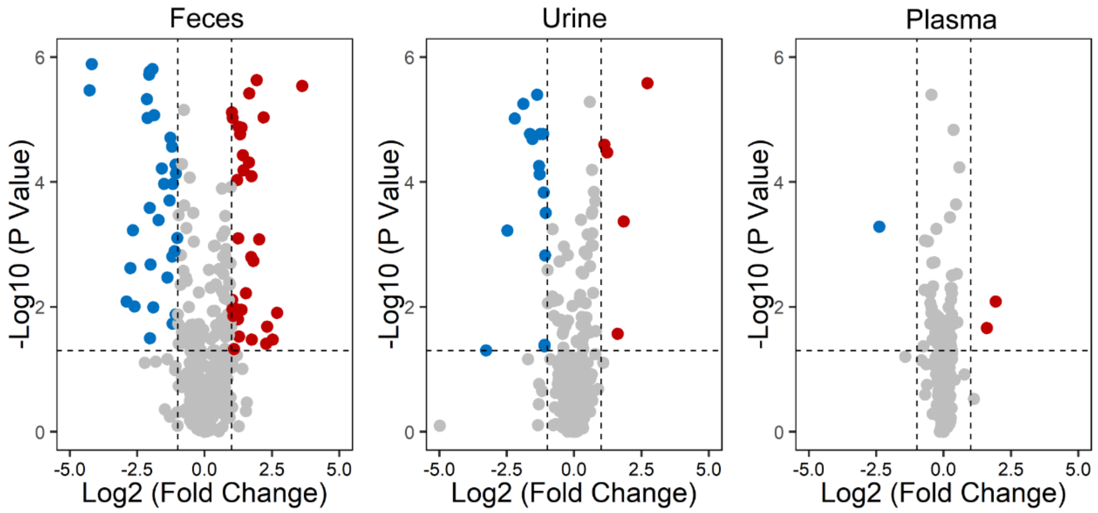

Figure S2. Volcano plots of feature changes of CIS versus control in feces, urine, and plasma. Each dot represents a metabolite identified in the sample. Blue dot represents a metabolite that is downregulated in the CIS. Red dot represents a metabolite that is upregulated in the CIS.

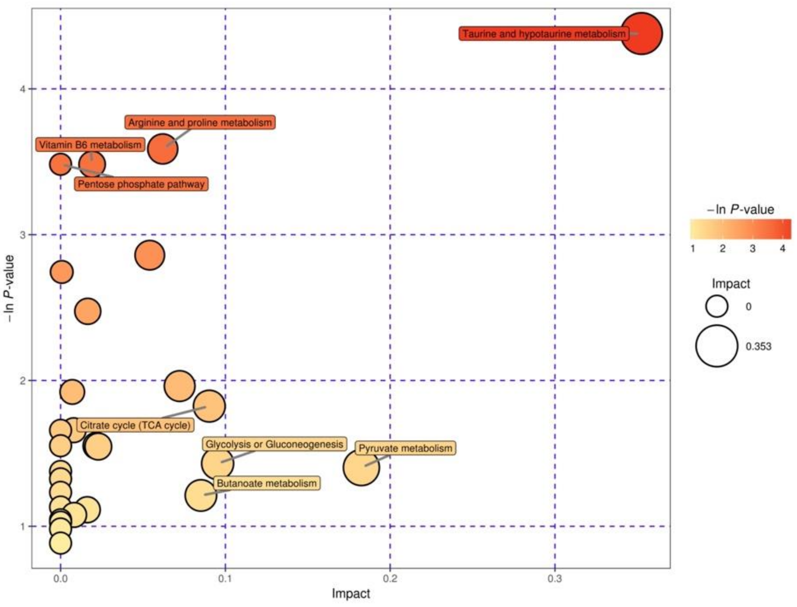

Figure S3. Metabolic pathway enrichment of differential metabolites in feces.

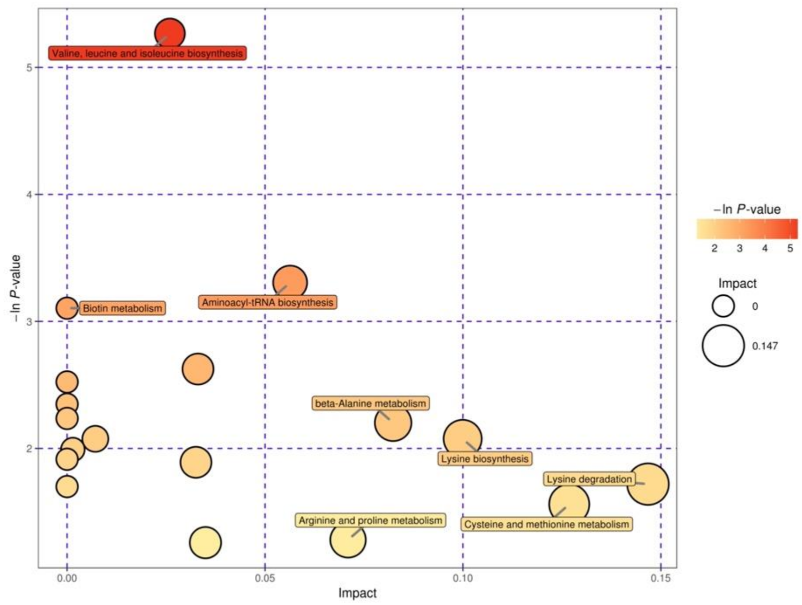

Figure S4. Metabolic pathway enrichment of differential metabolites in urine.

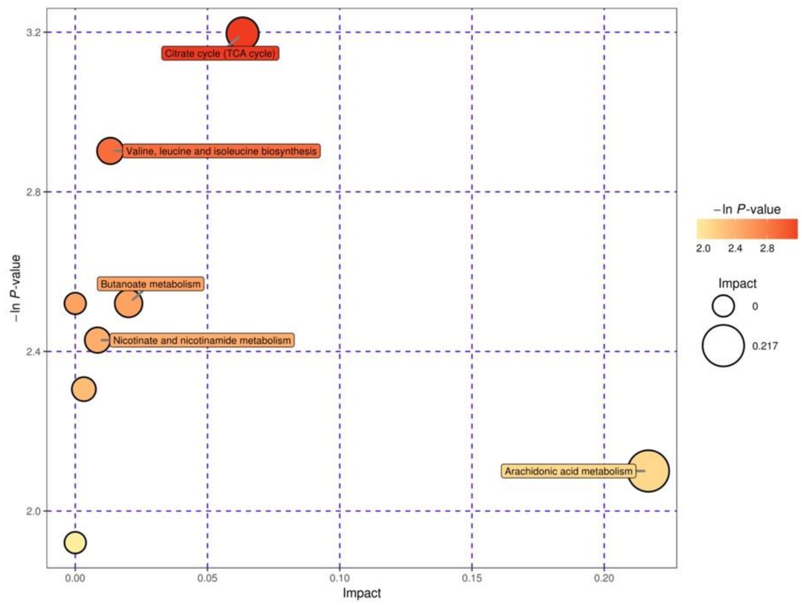

Figure S5. Metabolic pathway enrichment of differential metabolites in plasma.

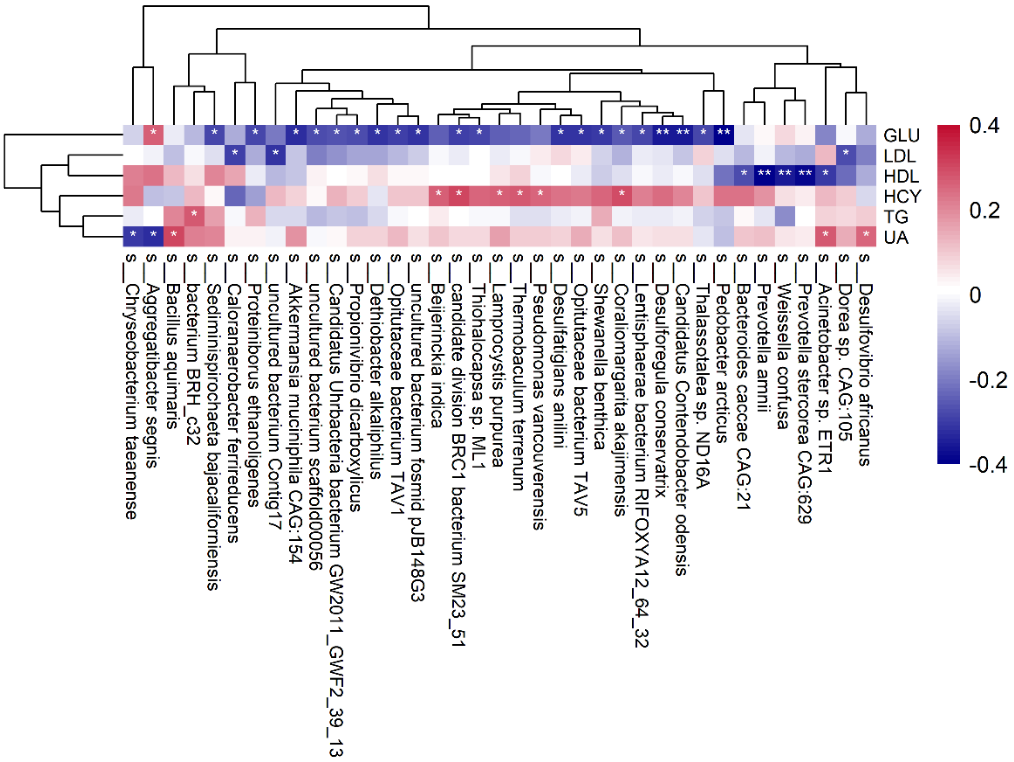

Figure S6. Hierarchical clustered heatmap of the Spearman's rank correlation coefficient of gut microbial species and blood clinical indexes. 222 pairs of correlations with 37 bacterial species and 6 blood clinical indices were plotted Red squares indicate positive associations between these microbial species and clinical indexes. Blue squares indicate negative associations. The statistical significance was denoted inside the squares (**P* < 0.05, ***P* < 0.01).

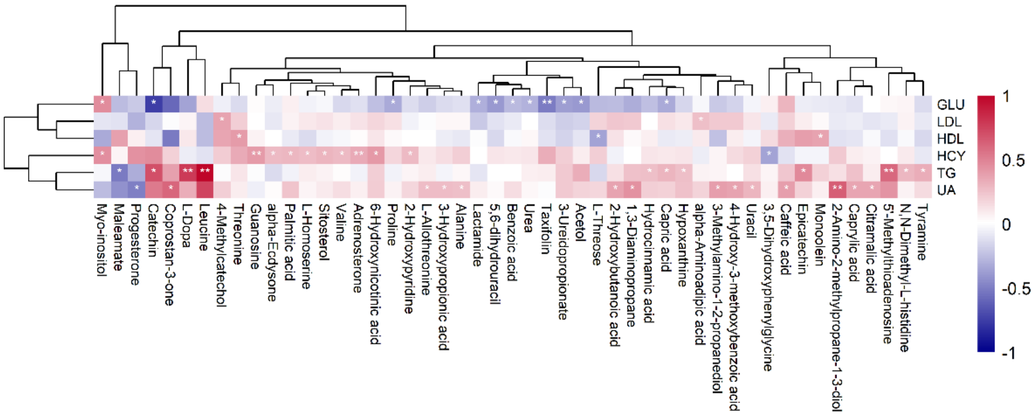

Figure S7. Hierarchical clustered heatmap of the Spearman's rank correlation coefficient of fecal metabolites and blood clinical indexes. 294 pairs of correlations with 49 fecal metabolites and 6 blood clinical indices were plotted. Red squares indicate positive associations between these microbial species and clinical indexes. Blue squares indicate negative associations. The statistical significance was denoted inside the squares (*P < 0.05, **P < 0.01).

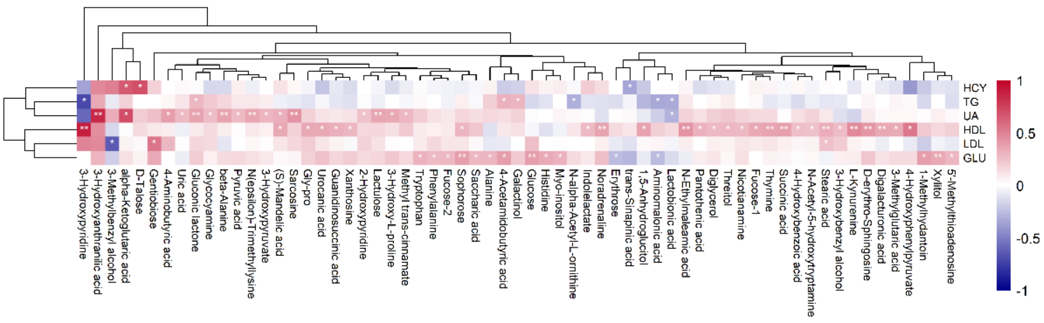

Figure S8. Hierarchical clustered heatmap of the Spearman's rank correlation coefficient of urinary metabolites and blood clinical indexes. 384 pairs of correlations with 64 urinary metabolites and 6 blood clinical indices were plotted. Red squares indicate positive associations between these microbial species and clinical indexes. Blue squares indicate negative associations. The statistical significance was denoted inside the squares (**P* < 0.05, ***P* < 0.01).

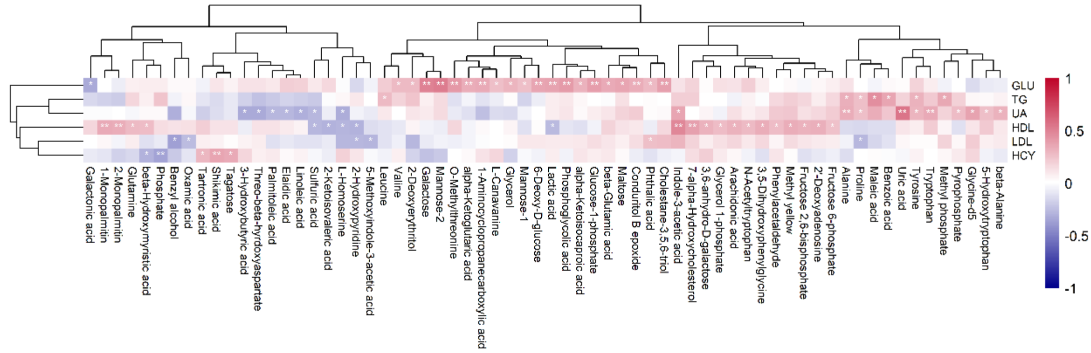

Figure S9. Hierarchical clustered heatmap of the Spearman's rank correlation coefficient of plasma metabolites and blood clinical indexes. 396 pairs of correlations with 66 plasma metabolites and 6 blood clinical indices were plotted. Red squares indicate positive associations between these microbial species and clinical indexes. Blue squares indicate negative associations. The statistical significance was denoted inside the squares (**P* < 0.05, ***P* < 0.01).

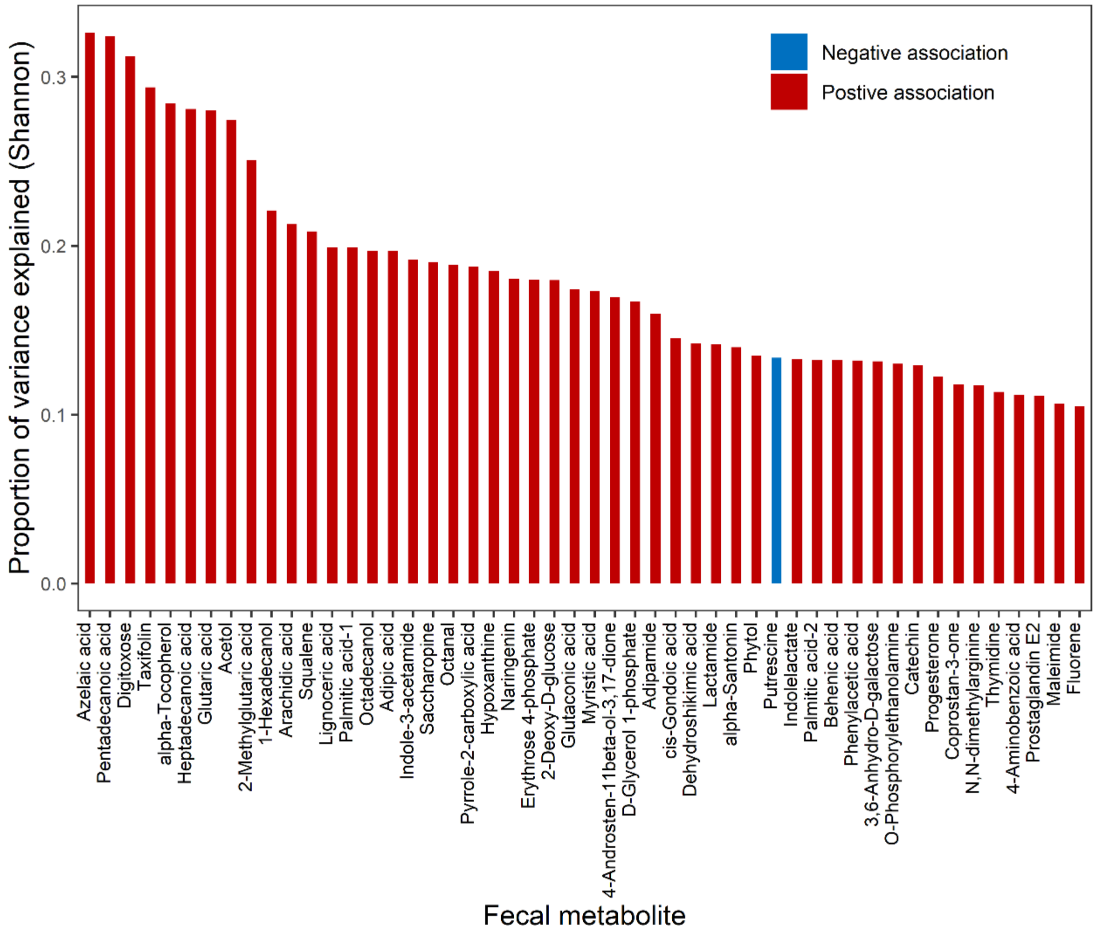

Figure S10. The proportion of variance in Shannon diversity that is explained by each fecal metabolite by the ordinary least square model. Red bar denotes positive associations between metabolite and Shannon diversity, while blue bar denotes negative associations.

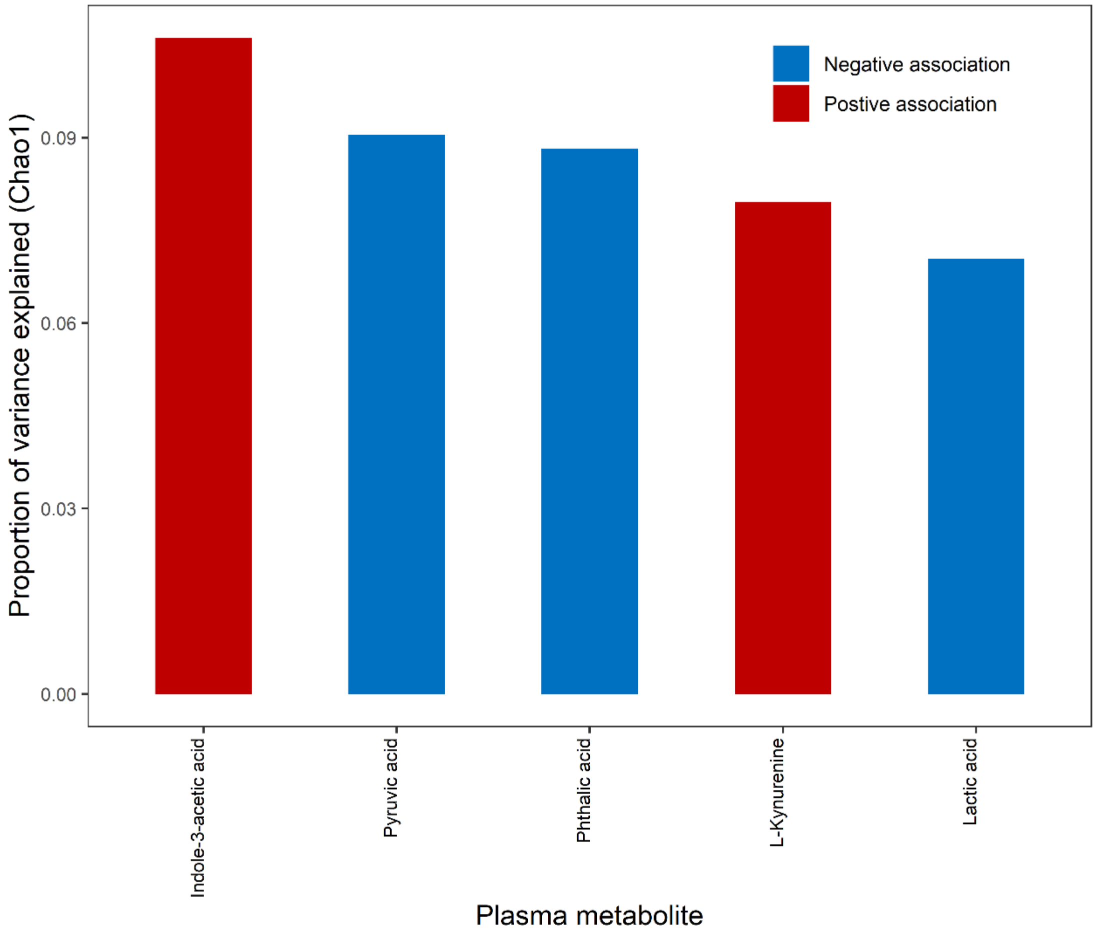

Figure S11. The proportion of variance in Chao1 diversity that is explained by each plasma metabolite by the ordinary least square model. Red bar denotes positive associations between metabolite and Chao1 diversity, while blue bar denotes negative associations.

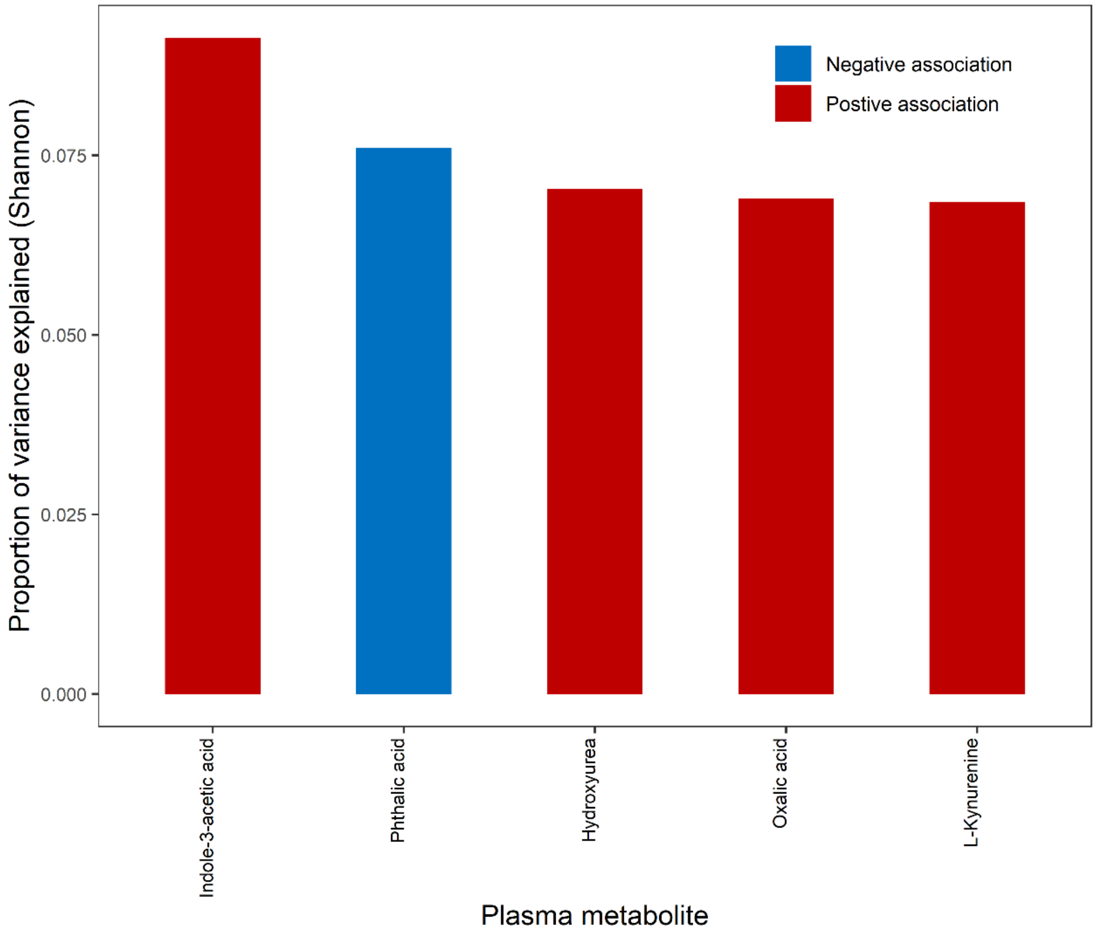

Figure S12. The proportion of variance in Shannon diversity that is explained by each plasma metabolite by the ordinary least square model. Red bar denotes positive associations between metabolite and Shannon diversity, while blue bar denotes negative associations.

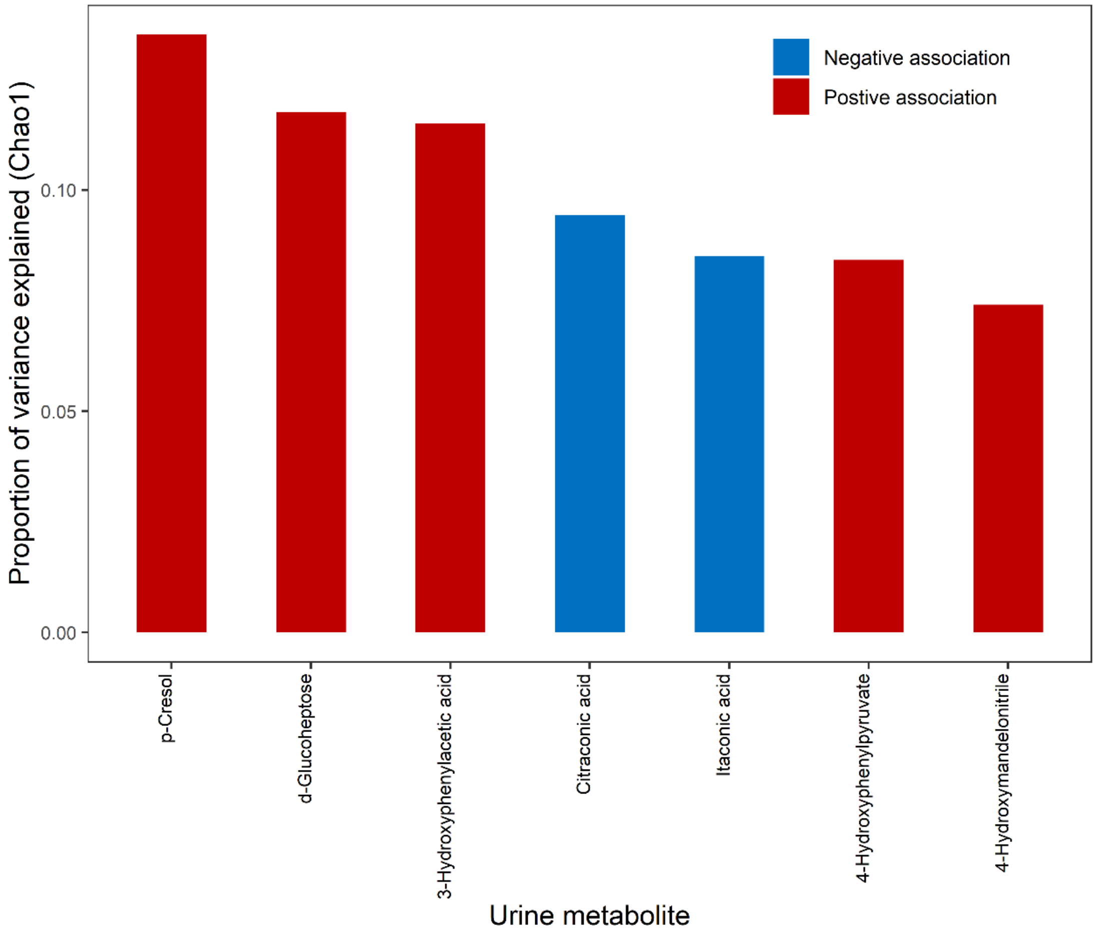

Figure S13. The proportion of variance in Chao1 diversity that is explained by each urine metabolite by the ordinary least square model. Red bar denotes positive associations between metabolite and Chao1 diversity, while blue bar denotes negative associations.

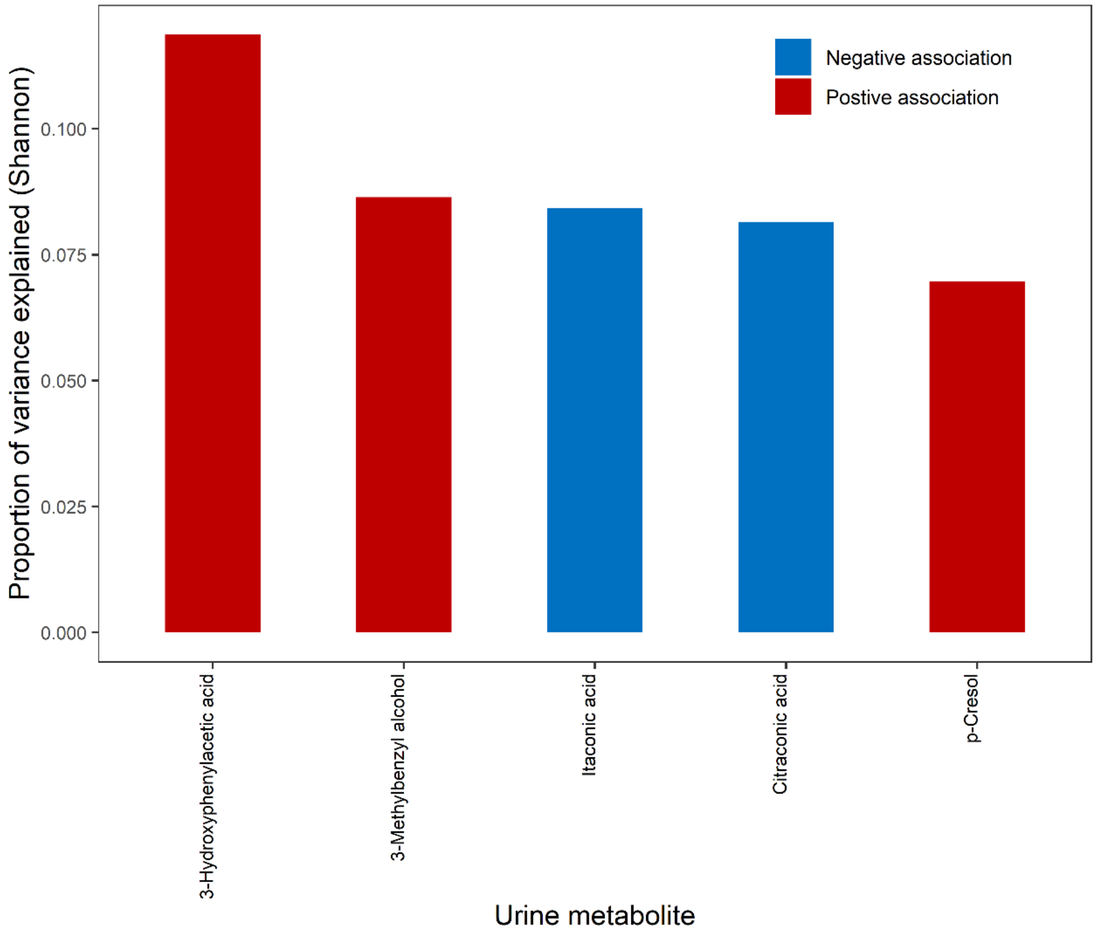

Figure S14. The proportion of variance in Shannon diversity that is explained by each urine metabolite by the ordinary least square model. Red bar denotes positive associations between metabolite and Shannon diversity, while blue bar denotes negative associations.

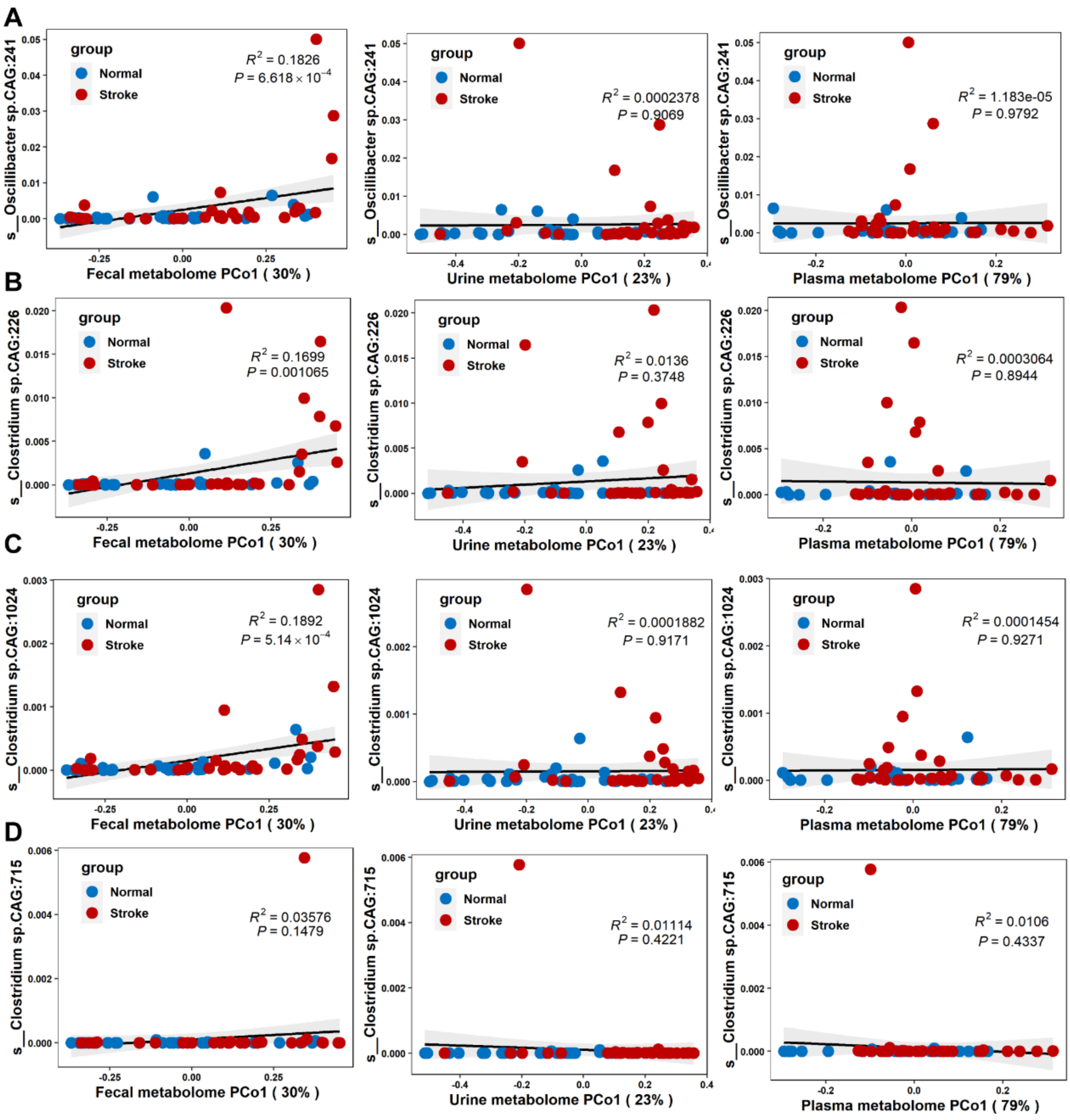

Figure S15. Correlation between bacteria data and the first principal coordinate (PCo1) of fecal, urine, and plasma metabolomics data. R2 and its significance were calculated using the ischemic stroke and control samples together. The black line and gray area show a linear model and its 95% confidence interval describing the overall trend. (A) Correlation between *Oscillibacter sp.CAG:241* and the first principal coordinate (PCo1) of fecal, urine, and plasma metabolomics data. (B) Correlation between *Clostridium sp.CAG:226* and the first principal coordinate (PCo1) of fecal, urine, and plasma metabolomics data. (C) Correlation between *Clostridium sp.CAG:1024* and the first principal coordinate (PCo1) of fecal, urine, and plasma metabolomics data. (D) Correlation between *Clostridium sp.CAG:715* and the first principal coordinate (PCo1) of fecal, urine, and plasma metabolomics data.

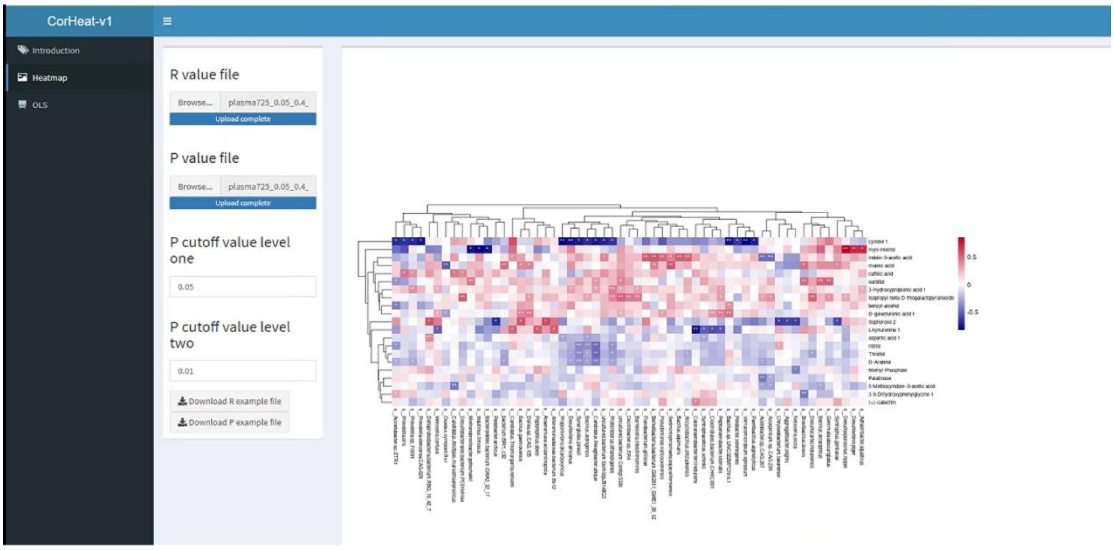

Figure S16. Showcase of association analysis on the CorHeat Lab web server

Table S1. Characteristics of the study participants and result of univariate logistic regression

Variable	Stroke(N=30)	Control(N=30)	*P*	OR
Gender Male	21(0.70)	18(0.60)	0.42	1.56
Age (years)	61(54-67)	62(55-69)	0.31	0.97
TG (mmol/L)	1.22(0.99-1.52)	1.35(1.08-1.59)	0.29	1.20
LDL (mmol/L)	2.20±0.68	2.31±0.50	0.51	0.74
HDL (mmol/L)	1.11±0.19	1.29±0.24	0.002**	0.02**
UA (umol/L)	303.80±64.50	282.30±62.68	0.20	1.01
Glu (mmol/L)	4.90(4.28-5.27)	5.06(4.80-5.58)	0.18	0.57
HCY (umol/L)	12.15(10.30-14.80)	11.6(10.40-13.90)	0.65	1.05

HDL: high-density lipoprotein, LDL: low-density lipoprotein, GLU: glucose of blood, UA: uric acid, TG: triglycerides, HCY: homocysteine. SD: standard deviation, OR: odds ratio, *P <=0.05, **P <=0.01. Continuous data are presented as mean±standard deviation or median (interquartile range). Categorical variables are presented as n (%).

Table S2. Metabolites that differ significantly in each metabolic sample

Metabolite	Fold change (FC)	Log2FC	*P*-value	Sample
p-Cresol	6.45	2.69	0.012	feces
Lactamide	4.98	2.32	0.021	feces
4-Hydroxyphenylacetic acid	4.84	2.27	0.039	feces
4-Hydroxy-3-methoxycinnamaldehyde	4.06	2.02	0.001	feces
Arbutin	3.81	1.93	0.000	feces
Saccharopine	3.34	1.74	0.000	feces
N-alpha-Acetyl-L-ornithine	3.13	1.65	0.000	feces
Phenylacetic acid	2.88	1.53	0.006	feces
Glutaraldehyde	2.58	1.36	0.011	feces
2-Hydroxybutanoic acid	2.41	1.27	0.030	feces
Octadecanol	2.34	1.23	0.016	feces
Thymine	2.18	1.12	0.012	feces
3-ureidopropionate	2.09	1.06	0.014	feces
Gentiobiose	0.49	-1.02	0.001	feces
trans-4-Hydroxy-L-proline	0.47	-1.08	0.013	feces
Epicatechin	0.47	-1.08	0.000	feces
Glucuronic acid	0.44	-1.19	0.000	feces
Fucose	0.44	-1.19	0.019	feces
3-Phenyllactic acid	0.44	-1.20	0.002	feces
4-Hydroxybenzaldehyde	0.43	-1.22	0.000	feces
2-5-Dihydroxybenzaldehyde	0.41	-1.27	0.000	feces
Estra-1-3-5(10)-triene-3-6beta-17beta-triol	0.27	-1.88	0.000	feces
Sophorose	0.27	-1.91	0.010	feces
Monoolein	0.25	-2.01	0.002	feces
Lactic acid	0.25	-2.03	0.032	feces
dl-p-Hydroxyphenyllactic acid	0.24	-2.04	0.000	feces
Progesterone	0.23	-2.12	0.000	feces
4-Aminobutyric acid	0.13	-2.90	0.008	feces
Diglycerol	0.05	-4.19	0.000	feces
Threonic acid	0.05	-4.27	0.000	feces
5-Aminovaleric acid lactam	6.60	2.72	0.000	urine
Cyclohexane-1-2-diol	3.06	1.62	0.027	urine
3-Methylbenzyl alcohol	2.17	1.12	0.000	urine
Noradrenaline	0.47	-1.10	0.043	urine
Citramalic acid	0.47	-1.10	0.041	urine
3-Hydroxybenzoic acid	0.34	-1.55	0.000	urine
Melezitose	0.27	-1.89	0.000	urine
Lactose	3.80	1.93	0.008	plasma
Sorbitol	3.03	1.60	0.022	plasma
N-Acetyl-beta-D-mannosamine	0.19	-2.39	0.001	plasma
